# Physics-Consistent Sparse Learning for Cross-Domain Rotor Fault Diagnosis

**DOI:** 10.3390/s26216686

**Published:** 2026-10-22

**Authors:** Anil Kumar, Chander Parkash, Emiliano Mucchi, Xiao Zhuang, Di Zhou, Zijian Qiao, Jiawei Xiang

**Affiliations:** 1 College of Mechanical and Electrical Engineering, Wenzhou University, Wenzhou 325035, China; zhuangxiao@wzu.edu.cn (X.Z.); wxw8627@163.com (J.X.); 2 Provincial Key Laboratory of Equipment Monitoring and Intelligent Operation & Maintenance Under Extreme Working Conditions, Wenzhou 325035, China; 3 Department of Mathematics, Patel Memorial National College, Rajpura 140104, India; cchanderr@gmail.com; 4 Department of Engineering, University of Ferrara, 44121 Ferrara, Italy; 5 Ningbo Key Laboratory of Micro-Nano Motion and Intelligent Control, Faculty of Mechanical Engineering and Mechanics, Ningbo University, Ningbo 315211, China; qiaozijian@nbu.edu.cn

**Keywords:** physics-guided deep learning, numerical modelling, rotor defect diagnosis, system identification, sparse domain adaptation

## Abstract

Robust rotor fault diagnosis is challenging when labeled fault data are limited and operating conditions differ between synthetic and real environments. Existing domain-adaptation methods can reduce distributional discrepancies, but they may retain redundant features and provide limited physical interpretability. This study proposes a physics-guided sparse-entropy domain adaptation framework for robust rotor fault diagnosis under such distribution shifts. Bearing eccentricity and damping are identified from experimental measurements and used to construct a physics-consistent rotor model. Synthetic fault data are then generated by incorporating representative defects, including unbalance and misalignment, while unlabeled signals, acquired from a laboratory rotor test rig, are used as the target domain. A ResNet-based feature extractor is trained using a hybrid strategy that combines labeled synthetic data with unlabeled experimental data. Maximum Mean Discrepancy and a Domain-Adversarial Neural Network are employed to reduce the source–target distribution discrepancy. In addition, sparsity and entropy regularization are incorporated into the objective function to suppress redundant representations and enhance fault-discriminative features. A Monte Carlo simulation-based optimization strategy is used to balance classification, domain alignment, and sparse–entropy constraints. Experimental results demonstrate an average target-domain accuracy of 94.04%, with a transfer gap of only 5.16%. The proposed framework also achieves higher diagnostic accuracy than DANN, Correlation Alignment, and MMD-based approaches. These results demonstrate its potential for data-efficient and physically interpretable rotor condition monitoring under varying operating conditions.

## 1. Introduction

A rotor is a shaft-mounted rotating component in machines [1,2]. It is a fundamental component of various types of machinery, including electric motors, turbines, and pumps [3,4], where it plays a vital role in energy conversion, power generation, and fluid transport. The rotor’s condition directly affects the overall performance, operational efficiency, and reliability of these machines [5,6]. The reliability and efficiency of rotating machinery are crucial to the operational success of numerous industrial applications, ranging from power generation and manufacturing [7] to the aerospace and automotive sectors [8]. Ensuring the smooth functioning of these machines necessitates the prompt identification and rectification of common defects, such as rotor unbalance and misalignment. If left unaddressed, these defects can lead to significant issues, including increased vibrations, excessive wear, energy losses, and, ultimately, catastrophic failure of the machinery.

Unbalance in rotors occurs when there is an uneven mass distribution, causing the rotor to exert uneven centrifugal forces during rotation [9,10]. This imbalance leads to vibrations and additional stresses on bearings and other components, potentially reducing the machinery’s lifespan. Misalignment [11,12], on the other hand, refers to the condition where the rotational axes of interconnected components do not align properly. This misalignment can stem from improper installation, thermal expansion, or operational loads, and, like unbalance, it can cause severe vibrations, increased friction, and mechanical degradation.

Dynamic simulation techniques provide a sophisticated approach to identifying these defects in real time and under actual operating conditions. By creating detailed computational models that replicate the behavior of rotors, the effects of unbalance and misalignment can be predicted and analyzed. Deng et al. [13] proposed a rotor finite element mimetic neural network (RFEMNN) that effectively combines physics-informed deep learning with finite element methods to accurately diagnose rotor faults, such as shaft cracks and unbalance, achieving a comprehensive diagnostic accuracy of 94.7%. To monitor health conditions of a centrifugal pump, Lan et al. [14] developed a physics-guided monitoring framework that relies on PCA (principal component analysis), EMD (empirical mode decomposition), CAF (cyclic auto correlation function), and CNN. The article [15] suggests a force model that is related to inter-shaft bearings with outer/inner race defects on the surfaces and identifies the non-linear dynamic characteristics of a dual-rotor system affected by local defects. Using numerical simulation techniques, Tang et al. [16] effectively illustrated a dynamic model required for a rotor-bearing system and validated the experimental investigations for a misalignment-rubbing coupling defect of an axial piston pump. Song et al. [17] analyzed the unbalanced characteristics of axial-varying eccentric rotors fitted in permanent magnet synchronous motors. The authors described a correlation among various entities, including unbalanced magnetic pull (MP), magnetic flux, and vibration signals, by employing signal demodulation, experimental treatments, and existing 3D finite element analysis. Yang et al. [18] described the vibration-based modeling of a four-disk with a hollow flexible shaft rotor system fitted in an aero-engine. The authors established a simple rotor model and experimented with the vibration transmission of a four-disk hollow flexible shaft fitted in the rotor system. The research work [19] emphasized the importance of developing accurate models for diagnosing faulty behavior in cracked rotors and provides insights into the dynamic response of unbalanced, cracked rotors. They conducted finite element analysis, numerical simulations, and experimental verification to support their findings.

Wang et al. [20] carried out dynamic modeling to analyze a two-span rotor-pedestal system fitted with bearing tilt as well as an extended defect. The underlying work [21] focused on complex dynamic loads during the contact performance of support bearings fitted in a rotor-bearing system. Additionally, it can analyze the interactions between various components, such as the race and roller of bearings, dynamic rotor eccentricity, and external excitation under non-uniform loads induced by gear engagement. Zhao et al. [22] proposed a data-driven approach, verified through simulation and experimental results, that effectively assesses rotor rub-impact fault severity, outperforming traditional frequency and time-domain indicators. Baum et al. [23] successfully analyzed a rotor system fitted in air-lubricated plain and foil journal bearings. This study compares the behavior of a perfectly balanced rotor fitted in rigid and foil bearings, taking into consideration the factors like clearance, rotor mass, and compliance of the bearings’ foils. The studies reveal significant differences in stability thresholds, bifurcation scenarios, and minimal film heights between the two bearing types. Additionally, the impact of static unbalance and varying compliance of the foil structure on the system’s dynamic behavior is discussed. While working on vertical rotor systems, Mohammad A. AL-Shudeifat [24] experienced the impact of non-synchronous whirl on the breathing mechanism of transverse cracks. The underlying study discovers the activation of the crack breathing mechanism during passage through resonance rotational speeds and its association with the excitation of post-resonance backward whirl phenomena.

Zhao et al. [25] introduced a different methodology for understanding transient rotor dynamic balancing. The method involves calculating excitation forces and identifying unbalanced parameters to balance the rotor system during acceleration. Numerical simulations and experimental treatments were employed in order to justify the accuracy as well as efficiency of the propounded method. Filsoof et al. [26] demonstrated a novel methodology to analyze the modeling properties of MRWTs (Multi-Rotor Wind Turbines) and represented a technical framework for establishing a high-fidelity linear time-invariant model of MRWTs. Sun et al. [27] developed a novel physics-guided KE (knowledge guided) framework for improving condition monitoring under pre-defined trained data conditions and low SNR, intended to measure blade conditions of wind turbine. By employing the existing improved time-varying mesh stiffness model, Yang et al. [28] demonstrated a dynamic analysis of a spur-geared rotor system with tooth tip chipping. Zhang et al. [29] developed an improved quasi-static model to study the effects of ring misalignment on ball bearings and rotor systems. The model under study can measure contact angles and determine the ball-raceway contact state.

Despite significant advancements in rotor condition monitoring, critical research gaps persist in achieving robust real-world performance. While laboratory-developed methodologies and dynamic models effectively diagnose common defects, such as unbalance and misalignment, they face substantial limitations in practical settings. Many existing approaches rely on complex models and extensive data, resulting in high computational demands and reduced efficiency. Crucially, a significant simulation-to-reality gap undermines methods that depend on physics-based simulations. Although these simulations offer a cost-effective way to generate labeled synthetic fault data, inherent discrepancies between simulated and real-world data degrade the performance of models trained solely on synthetic information, especially under domain shift where real labeled data is scarce. Liu et al. [30] developed a novel transfer learning network-based domain adaptation methodology that can enhance defect identification accuracy and reduce domain differences, caused by complicated machine components, for different gas turbines working under varying working environments. Recent studies have explored deep domain adaptation (DA) techniques to reduce the discrepancy between simulated and experimental data. For example, Domain-Adversarial Neural Networks (DANN) learn domain-invariant features [31], while feature selection methods identify fault-relevant features [32]. However, these approaches do not necessarily ensure that the aligned features are the most informative for fault diagnosis or retain clear physical interpretability. Therefore, an effective framework is needed to align data across domains while prioritizing fault-discriminative and physically interpretable features.

However, these approaches mainly focus on reducing distribution discrepancies and may not explicitly control redundant or less informative feature representations. This limitation is particularly relevant to rotor systems, where domain shifts can be significant and fault signatures may be subtle. Although sparsity-based learning has shown potential for selecting informative features and improving feature interpretability, its application within physics-guided domain adaptation for rotor fault diagnosis remains insufficiently explored. These gaps motivate the development of a framework that combines physics-guided synthetic data generation, domain adaptation, and sparsity–entropy-based feature learning for robust diagnosis under limited labeled experimental data.

To overcome the above-mentioned interconnected challenges, the underlying study propounds a novel sparsity-enabled physics-guided machine learning framework. The approach integrates system identification to extract key modal parameters (unbalance and bearing damping) for constructing a high-fidelity numerical rotor model, which is then used to simulate diverse fault scenarios and generate comprehensive labeled synthetic data. Concurrently, unlabeled real vibration data is collected from a physical test rig. A hybrid deep learning model, based on a Residual Network (ResNet), is trained on both data domains. Domain adaptation is achieved by jointly employing Maximum Mean Discrepancy (MMD) and DANN to minimize distributional differences. To address the core limitation of uniform feature alignment, an entropy-based sparsity regularization term is introduced into the domain adaptation loss, compelling the network to focus on the most discriminative and interpretable fault features while discarding redundant or noisy information. A Monte Carlo-based optimization strategy fine-tunes the regularization parameters, ensuring a balanced trade-off between domain adaptation effectiveness and feature selectivity. The framework leverages physics-based modeling to guide feature generation, employs domain adaptation to bridge source–target discrepancies, and applies sparsity-driven regularization to isolate fault-critical signatures, thereby delivering superior accuracy, robustness, and interpretability in rotor fault diagnosis.

The novelty of the proposed framework lies in extending conventional domain adaptation beyond distribution alignment by incorporating selective feature learning under physical guidance. The sparsity–entropy constraint suppresses redundant features and enhances fault-relevant information, while the physics-guided source domain reduces the dependence on labeled experimental fault data. This combination provides a unified solution to domain discrepancy, feature redundancy, and limited labeled data.

The offered study can be structured as follows. Section 2 presents the foundational concepts, including deep learning and domain adaptation theory for system identification. Section 3 outlines the proposed methodology. Section 4 presents the application of the approach, covering dynamic modeling, system identification for parameter optimization, defect analysis using dynamic simulation and experimental validation, physics-guided modeling, and a comparative evaluation with state-of-the-art methods. Finally, Section 5 concludes the study with key insights and future research directions.

## 2. Theory

This section presents the theoretical foundations relevant to this study, including key concepts of deep learning, theory of domain adaptation, and principles of system identification.

### 2.1. Convolutional Neural Network (CNN)

One-dimensional CNN is a deep learning model especially designed for processing the sequential data with one-dimensional input. This type of CNN is commonly used for forecasting time series and some tasks like classification, signal processing, and natural language processing.

#### Convolution

Given an input sequence $x=[x_{1},x_{2},\ldots ,x_{K}]$ and a kernel *w* = [$w_{1},w_{2},\ldots ,w$]; the convolution operation is defined by Equation (1) [33,34,35]:

(1) $$y\left(t\right)=\sum_{k=1}^{K}w_{k}x_{t+k-1}$$

where y(*t*) is the output at time step t, and *K* is the kernel size.

Following the convolution operation, an activation function called ReLU (Rectified Linear Unit) and defined by Equation (2) is applied. The convolution operation can introduce non-linearity into the model and thus allows it to understand and capture complex patterns more effectively:

ReLU(*z*) = max (0, *z*) (2)

Pooling layers, such as max pooling, are often applied to reduce the dimensionality of the data, retaining essential features while discarding less relevant information. For instance, max pooling with a pool size of 2 reduces the size of the feature map by half.

### 2.2. Domain Adaptation

Domain adaptation, a branch of transfer learning, addresses performance drops that occur when training (source) and testing (target) data follow different distributions. To handle this, we adopt a Domain-Adversarial Neural Networks (DANN), where simulation data act as the source domain, whereas experimental data act as the target domain. DANN jointly learns features that are discriminative for classification and invariant to domain shifts. A signal encoder extracts shared representations, while a domain discriminator distinguishes between domains. Using a Gradient Reversal Layer, the feature extractor is trained adversarial to deceive the discriminator, resulting in domain-invariant features that generalize well across both domains [36,37,38].

The training objective in DANN incorporates multiple loss components to jointly achieve accurate classification on the source domain and effective domain adaptation to the target domain. The first term is the classification loss, computed using the cross-entropy loss over labeled source samples (Equation (3)):

(3) $$L_{class}=-\frac{1}{N_{s}}\sum_{i=1}^{Ns}\sum_{c=1}^{C}y_{i,c,}log(p_{i,c,})$$

where Ns denotes the number of source samples, C is the number of classes, and $y_{i,c}\;$is the true predicted probability of class c for sample i.

The second component is the domain adversarial loss, which is designed to align the feature distributions between the source and target domains. It is defined by Equation (4):

(4) $$L_{domain}=-\frac{1}{N_{s}+N_{t}}[\sum_{i=1}^{Ns}log(D(f_{i}))+\sum_{i=1}^{Nt}log(1-D(f_{i}))$$

where $N_{t}$ denotes the number of target samples, $D(f_{,})$ is the output of the domain discriminator (probability that feature f is from the source), and $f_{i,}$ $f_{j}$ are features from the encoder for source and target samples, respectively. This loss is optimized adversarialy using a Gradient Reversal Layer (GRL), which multiplies the gradients by −λ, evolving with the training progress.

To further reduce domain shift, the model may include the MMD loss (Equation (5)), which measures the discrepancy between source and target feature distributions in a reproducing kernel Hilbert space (RKHS):

(5) $${L_{MMD}=MMD}^{2}(Xs,Xt)={∥\mu s\;-\mu t\;∥}_{H}^{2}$$

where ${∥\cdot ∥}_{H}$ denotes the norm in the RKHS.

The empirical mean embedding (EME) of the two domains (source and target) can be defined by Equations (6) and (7).

(6) $$\mu s=\frac{1}{n}\sum_{i=1}^{n}\varphi (x_{si})$$

(7) $$\mu t=\frac{1}{n}\sum_{i=1}^{n}\varphi (x_{ti})$$

where φ(⋅) denotes the feature function that can map each sample to the RKHS.

**Figure 1:**
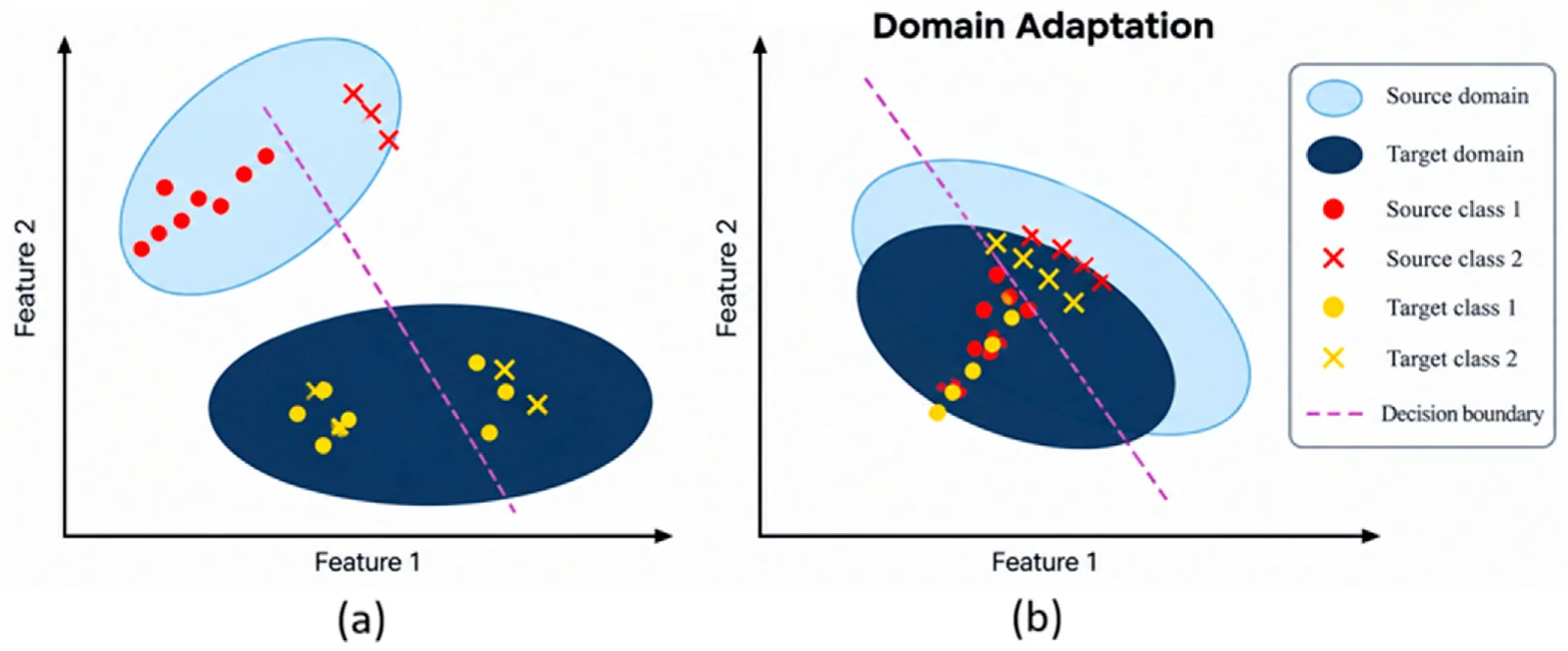
Principle of MMD (**a**) before domain adaptation and (**b**) after domain adaptation.

The principle of MMD is well described in Figure 1. The MMD method can be implemented as described in the research [39,40,41,42].

The term $L_{entropy}$ represents an entropy-based regularization (Equation (8)), which is applied across the layers of a deep neural network. It computes the Shannon entropy of the normalized absolute weight magnitudes within each layer, treating them as a probability distribution. By penalizing high entropy, this term encourages a sparse distribution of weights, where only a few connections carry significant importance. Such sparsity promotes model interpretability, reduces over-fitting, and improves computational efficiency. In essence, this regularization guides the network to focus on the most informative parameters by discouraging uniformly distributed or redundant weight configurations.

(8) $$L_{entropy}=-\sum_{l=1}^{L}\sum_{i,j}\frac{\left|w_{i,j}^{l}\right|}{\sum \left|w^{l}\right|}\log\frac{\left|w_{i,j}^{l}\right|}{\sum \left|w^{l}\right|}$$

Finally, the overall objective function is expressed in Equation (9):

(9) $$L_{total}=L_{class}+\;L_{domain}+{\lambda_{1}L}_{MMD}+\lambda_{2}L_{entropy}$$

where $L_{class}$ denotes the classification loss, $L_{domain}$ represents the domain-discrimination loss, $L_{entropy}$ denotes the weight-entropy regularization term, and $L_{MMD}$ represents the MMD loss. The parameters $\lambda_{1}$, and $\lambda_{2}$ determine the contributions of the coresponding term.

**Figure 2:**
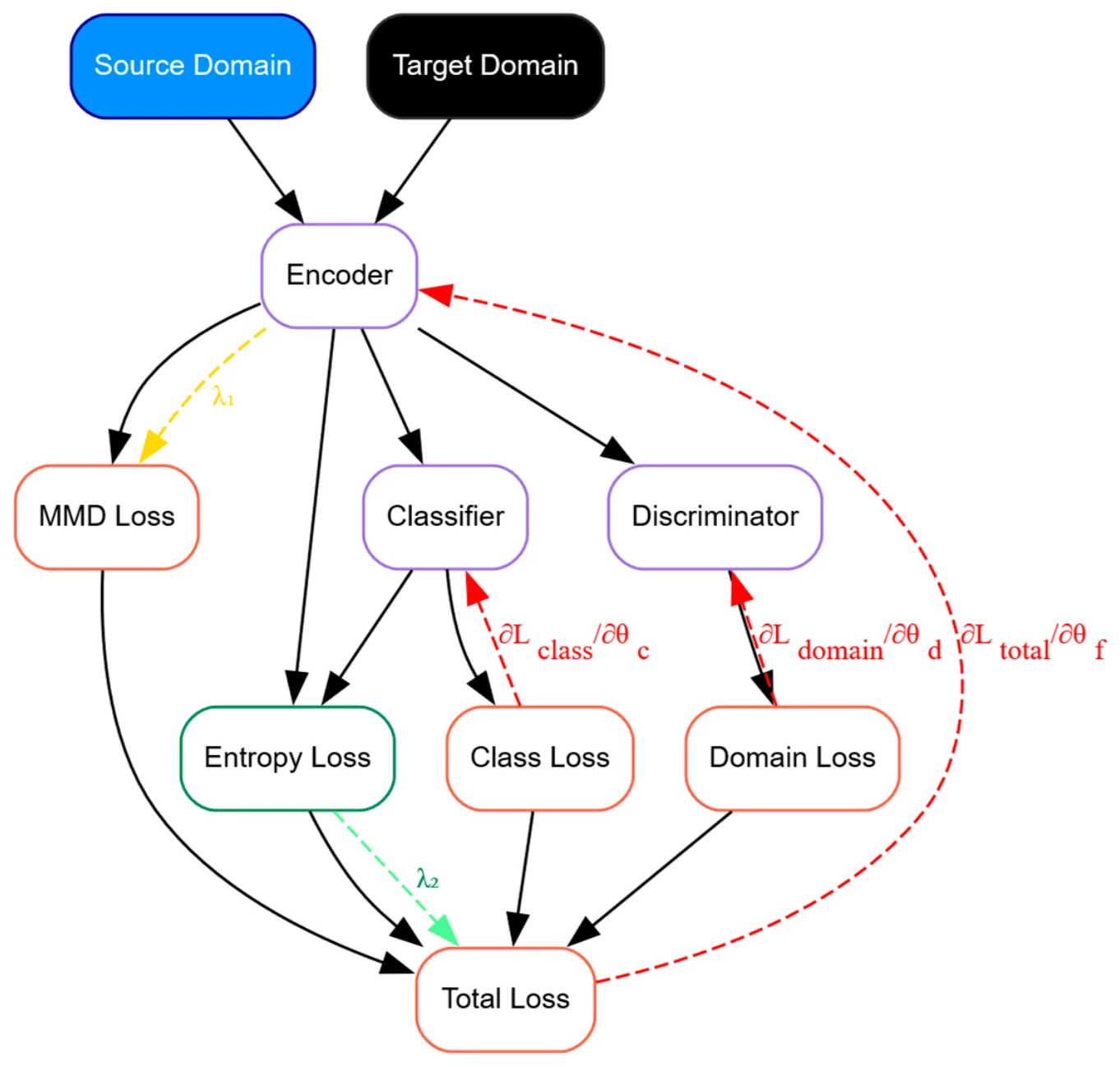
Working of sparsity-based domain adaptation.

Figure 2 illustrates the schematic representation of how the various loss components are computed and utilized to update different parts of the network. For example, the total loss is used to update the feature extractor. The classification loss ($L_{class}$) is used to update the classifier ($\theta_{c}$). The domain loss is used to train the domain discriminator and simultaneously guide the feature extractor ($\theta_{f}$) to learn domain-invariant features. The MMD loss ($L_{MMD}$) aligns the distributions of the source and target domains in the feature space, while the entropy loss ($L_{entropy}$) encourages sparsity for confident predictions on the target data, and domain loss ($L_{domain}$) is used to update the discriminator ($\theta_{d}$).

### 2.3. System Identification

The term “System identification” is a structured methodology used to derive a mathematical model of a physical phenomenon (system) from its time-series data. The procedure is initiated by collecting and preparing of input-output data, followed by the selection of a suitable model structure. An initial parameter estimate is then refined through optimization, where an objective function (OF) is typically designed by minimizing the sum of squared errors between the measured and predicted outputs and is leveraged by an algorithm like the Levenberg–Marquardt method to iteratively adjust the parameters. This iterative minimization of prediction error culminates in a tuned model that reliably captures the system’s core dynamics. The system identification is carried out in the following steps:

**Step 1:** Acquire and preprocess high-frequency time-series displacement measurements of the system in both directions (horizontal (x) and vertical (y) (Equation (10)).

**Step 2:** Generate initial estimates of the model parameter (θ), employing empirical engineering methods.

**Step 3:** Establish the objective function (OF), employing a cost function based on cosine similarity.

(10) $$\mathrm{Let}v_{meas}^{\left(i\right)}=\left[\begin{array}{c} x_{meas}^{\left(i\right)}\\ y_{meas}^{\left(i\right)} \end{array}\right]\;\mathrm{and}\;v_{model}^{\left(i\right)}=\left[\begin{array}{c} {x_{model}(t,\theta )}^{\left(i\right)}\\ {y_{model}(t,\theta )}^{\left(i\right)} \end{array}\right]$$

(11) $$cossin\left(\theta \right)=\frac{1}{N}\sum_{i=1}^{N}\frac{v_{meas}^{\left(i\right)}.v_{model}^{\left(i\right)}}{\left\|v_{meas}^{\left(i\right)}\right\|.\left\|v_{model}^{\left(i\right)}\right\|}$$

Then the loss function

(12) $$L\left(\theta \right)=1-cossin\left(\theta \right)$$

So, the system identification optimization becomes the following:

(13) $$\theta^{*}=\arg\;\begin{array}{c} min\\ \theta \end{array}L\left(\theta \right)$$

where $y_{meas}$—measured output in the vertical direction, $x_{meas}$—measured output in the horizontal direction, respectively. Moreover, $y_{model}(t,\theta )$—predicted output from the model in the vertical direction, and $x_{model}(t,\theta )$—predicted output from the model in the horizontal direction, respectively, at time t and model parameters θ.

**Step 4**: Predictions are generated using the current set of model parameters θ.

**Step 5**: An optimization procedure is applied to reduce the value of the defined objective (loss) function.

**Step 6**: The gradients of the loss function (Equation (12)) are calculated with respect to the parameters *θ*.

**Step 7**: The model parameters are updated according to the gradient descent rule.

(14) $$\theta_{new}=\theta_{old}-\mathrm{learning}\;\mathrm{rate}\;(\lambda )*\mathrm{gradients}\;(𝛻J(\theta )).$$

**Step 8**: The refined model parameters are evaluated on the validation dataset to verify the performance.

## 3. Methodology

**Figure 3:**
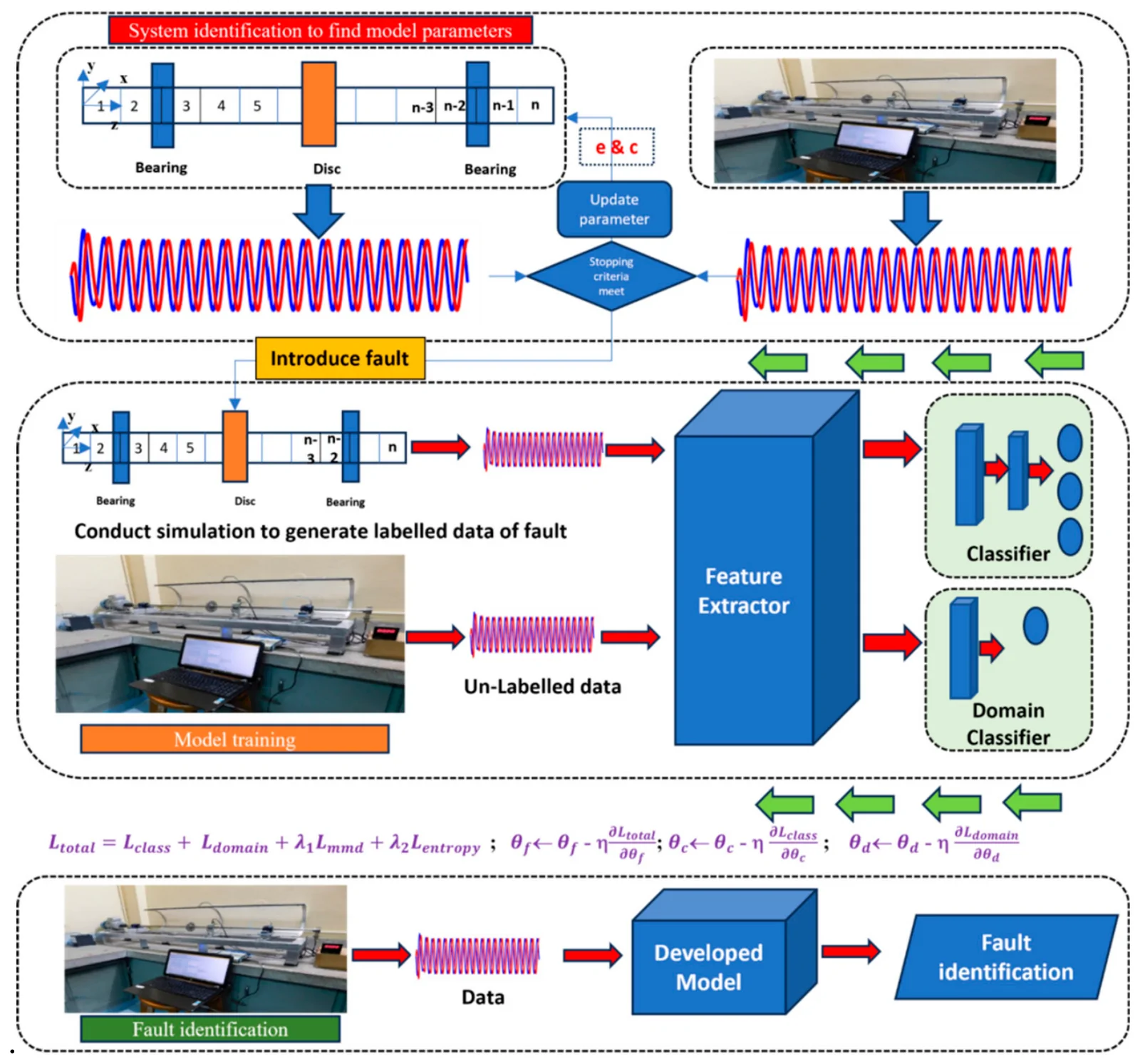
Layout of the methodology.

The detailed architecture of the proposed methodology is illustrated in Figure 3. The proposed framework integrates physics-based rotor modeling, numerical simulation, deep learning, and domain adaptation to bridge the discrepancy between simulated and experimental vibration data. The methodology consists of the following sequential steps.

**Step 1:** A numerical model of the defect-free rotor is established based on the governing rotor dynamic equations and physical parameters.

**Step 2:** The uncertain parameters of the numerical model, such as eccentricity and damping factor, are optimized by comparing the simulated vibration responses with experimentally measured responses and minimizing the corresponding response discrepancy.

**Step 3:** After validating the defect-free model, different rotor fault conditions, including unbalance and misalignment, are introduced by modifying the corresponding excitation and dynamic parameters.

**Step 4:** The validated numerical model is used to simulate vibration responses under different rotor operating and fault conditions, thereby generating labeled source-domain data.

**Step 5:** In parallel, vibration signals are acquired from the experimental rotor test rig. These experimental signals constitute the target domain and are treated as unlabeled data during model training. **Points** from the horizontal (X) and vertical (Y) directions are used as model inputs.

**Step 6:** The simulated and experimental vibration signals are processed for training, validation, and testing. The source-domain data were divided into 80% training and 20% validation, while the experimental target-domain data were divided into 70% unlabeled alignment data and 30% independent test data. The target-domain alignment data were used only for domain adaptation without using their class labels, whereas the held-out 30% target-domain data were used exclusively for final performance evaluation.

**Step 7:** The labeled source-domain data and unlabeled target-domain data are supplied to the proposed deep domain-adaptation network.

**Figure 4:**
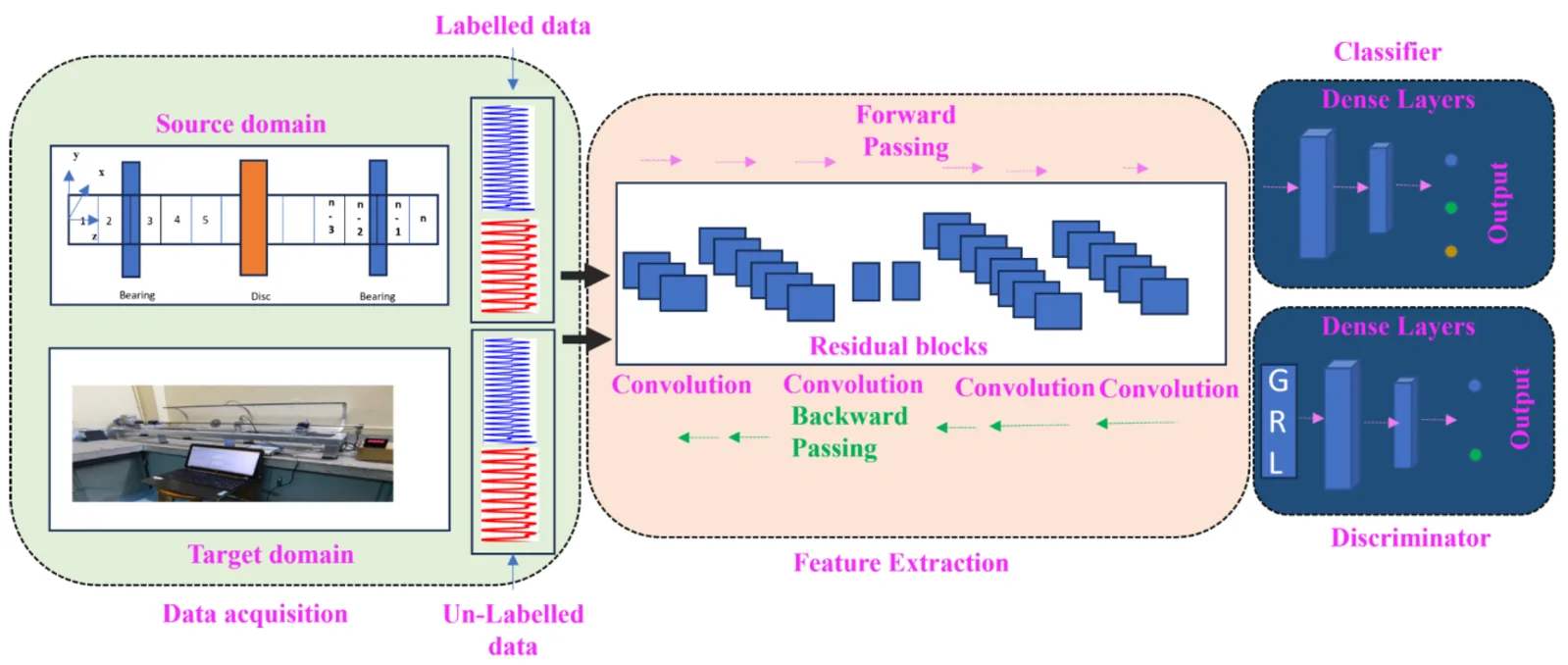
Detailed structure of the ResNet model used in this work.

**Step 8:** A ResNet-based feature extractor (Figure 4), consisting of convolutional and residual blocks, is employed to extract representative vibration features from the input signals.

**Step 9:** The extracted features are simultaneously supplied to a fault classifier and a domain discriminator. The classifier predicts the rotor condition, whereas the discriminator determines whether the extracted feature originates from the source or target domain. A gradient reversal layer (GRL) is introduced between the feature extractor and domain discriminator to promote the learning of domain-invariant features. Maximum Mean Discrepancy (MMD) is employed to further minimize the statistical discrepancy between the source-domain and target-domain feature distributions.

**Step 10:** A weight-entropy regularization term is incorporated to promote sparse and generalizable network representations.

**Step 11:** The complete training objective is formulated by combining the classification loss, domain-adversarial loss, weight-entropy regularization, and MMD loss.

**Step 12:** The network is trained using the Adam optimizer, with a learning rate of 0.001 and a batch size of 32. Labeled source-domain data are used to optimize the fault-classification branch, while source-domain and unlabeled target-domain data are jointly used for domain alignment.

**Step 13:** After training convergence, finally, unseen experimental vibration signals are provided to the trained network for fault identification.

## 4. Dynamic Modeling and Experimental Investigation

### 4.1. Dynamic Modeling

**Figure 5:**
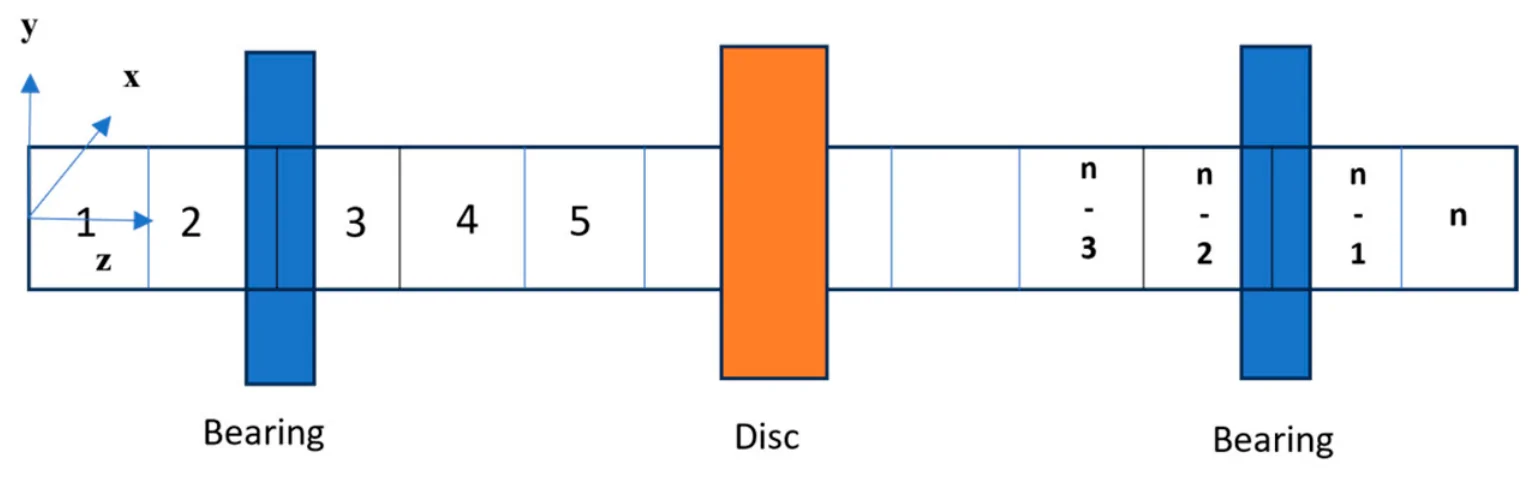
Distribution of elements.

The shaft is discretized using the Euler–Bernoulli beam element [43]. The length-to-diameter ratio is approximately 1.4/0.01552 = 90.2, indicating that the beam is slender. This justifies the assumption that plane sections remain plane and normal to the beam axis after deformation, thereby neglecting shear deformation. Each node in the finite element model has four DOFs (degrees of freedom): (i) translational displacements ($u_{x}$, $u_{y}$) in the lateral directions and (ii) rotational displacements ($\theta_{x}$, $\theta_{y}$) about the axes perpendicular to the beam axis. The element stiffness matrix is derived from beam bending theory and depends on the material’s Young’s modulus and the cross-sectional moment of inertia. The consistent mass matrix, which provides a more accurate representation of inertial effects than lumped mass approaches, is derived from the kinetic energy (KE) of the beam element. The assembly of these element matrices into global system matrices follows standard finite element procedures, with proper consideration of boundary conditions. The distribution of elements is represented as shown in Figure 5. The second-order differential Equation (15) is utilized to describe the dynamics of a rotor-bearing system [13].

(15) $$M\ddot{q}+(C+\omega G)\dot{q}+Kq=F(t)$$

where

M—Global mass matrix (includes rotor and disc masses)

C—Damping matrix (bearing damping + material damping)

K—Stiffness matrix (shaft stiffness + bearing stiffness)

ω—Rotational speed (rad/s)

q—Displacement vector [$u_{x}$,$u_{y}$,$\theta_{x}$,$\theta_{y}$] per node

F(*t*)—External forces (unbalance, misalignment, etc.)

The analysis is based on several key assumptions. First, small deformations are assumed, meaning that the shaft exhibits linear elastic behavior, with displacements and strains remaining sufficiently small to maintain a linear stress-strain relationship. Second, shear deformation is considered negligible, implying that plane sections remain plane and perpendicular to the neutral axis after bending. Finally, the shaft material is assumed to be isotropic, exhibiting uniform mechanical properties in all directions.

For each beam element of length L_e_, the stiffness and mass matrices can easily be derived employing fundamental energy principles. The stiffness matrix K (Equation (16)) captures the element’s resistance to bending. It is constructed as a block-diagonal matrix to account for independent bending in the x-z and y-z planes:

(16) $$K=\left[\begin{array}{cc} K_{uu} & 0\\ 0 & K_{vv} \end{array}\right]$$

Here, $K_{uu}$ is stiffness that resists bending in the horizontal direction, (Equation (17)) and $K_{vv}$ is bending resistance about the vertical direction (Equation (18)).

(17) Kuu=EIyyLe3126Le−126Le6Le4Le2−6Le2Le2−12−6Le12−6Le 6Le2Le2−6Le4Le2

$K_{vv}$ is stiffness that resists bending in the *y*-axis, and $I_{xx}$ is bending resistance about the *x*-axis.

(18) Kvv=EIxxLe3126Le−126Le6Le4Le2−6Le2Le2−12−6Le12−6Le 6Le2Le2−6Le4Le2

The consistent mass matrix M_e_, which accurately distributes the element’s inertial properties, is given by Equation (19):

(19) $$M_{e}=\frac{\rho AL}{420}\left[\begin{array}{cccccccc} 156 & 22L_{e} & 54 & -13L_{e} & 0 & 0 & 0 & 0\\ 22L_{e} & 4{L_{e}}^{2} & 13L_{e} & -3{L_{e}}^{2} & 0 & 0 & 0 & 0\\ 54 & 13L_{e} & 156 & -22L_{e} & 0 & 0 & 0 & 0\\ -13L_{e} & -3{L_{e}}^{2} & -22L_{e} & 4{L_{e}}^{2} & 0 & 0 & 0 & 0\\ 0 & 0 & 0 & 0 & 156 & 22L_{e} & 54 & -13L_{e}\\ 0 & 0 & 0 & 0 & 22L_{e} & 4{L_{e}}^{2} & 13L_{e} & -3{L_{e}}^{2}\\ 0 & 0 & 0 & 0 & 54 & 13L_{e} & 156 & -22L_{e}\\ 0 & 0 & 0 & 0 & -13L_{e} & -3{L_{e}}^{2} & -22L_{e} & 4{L_{e}}^{2} \end{array}\right]$$

The cross-coupling matrix $G_{e}$, is given by Equation (20):

(20) $$G_{e}=\frac{\rho J_{p}}{30L_{e}}\left[\begin{array}{cccccccc} 0 & 36 & 0 & -3L_{e} & 0 & -36 & 0 & -3L_{e}\\ -36 & 0 & 3L_{e} & 0 & 36 & 0 & 3L_{e} & 0\\ 0 & -3L_{e} & 0 & 4{L_{e}}^{2} & 0 & 3L_{e} & 0 & -{L_{e}}^{2}\\ 3L_{e} & 0 & -4{L_{e}}^{2} & 0 & -3L_{e} & 0 & {L_{e}}^{2} & 0\\ 0 & -36 & 0 & 3L_{e} & 0 & 36 & 0 & 3L_{e}\\ 36 & 0 & -3L_{e} & 0 & -36 & 0 & -3L_{e} & 0\\ 0 & 3L_{e} & 0 & -{L_{e}}^{2} & 0 & -3L_{e} & 0 & 4{L_{e}}^{2}\\ 3L_{e} & 0 & {L_{e}}^{2} & 0 & -3L_{e} & 0 & -4{L_{e}}^{2} & 0 \end{array}\right]$$

For the bearing, a simplified viscous damping model is considered (Equation (21)):

(21) $$F_{bearing}={-c}_{bearing}\dot{q}$$

The damping of each element is modeled using the proportional damping approach (Equation (22)).

(22) $$C_{e}=\alpha M+\beta K\;$$

where α and β are constants (α, β = 0.01).

The finite element formulation requires assembling element matrices (Ke, Me, Ge) into global matrices (K, M, G, C) for solving system-level equations of motion, and for a rotor discretised into N beam elements, each node has four DOFs,${\;q}_{i}={\left[u_{i},v_{i},\theta_{xi},\theta_{yi}\right]}^{T}$. Global K, Global M, and Global G are constructed by summing contributions from all elements (Equation (23)):

$$K=\begin{array}{c} N\\ ⨁\\ e=1 \end{array}K_{e},$$

(23) $$M=\begin{array}{c} N\\ ⨁\\ e=1 \end{array}M_{e}$$

$$G=\begin{array}{c} N\\ ⨁\\ e=1 \end{array}G_{e}$$

where ⊕ denotes addition (mapped via nodal connectivity).

### 4.2. Establishment of the Dynamic Model

**Figure 6:**
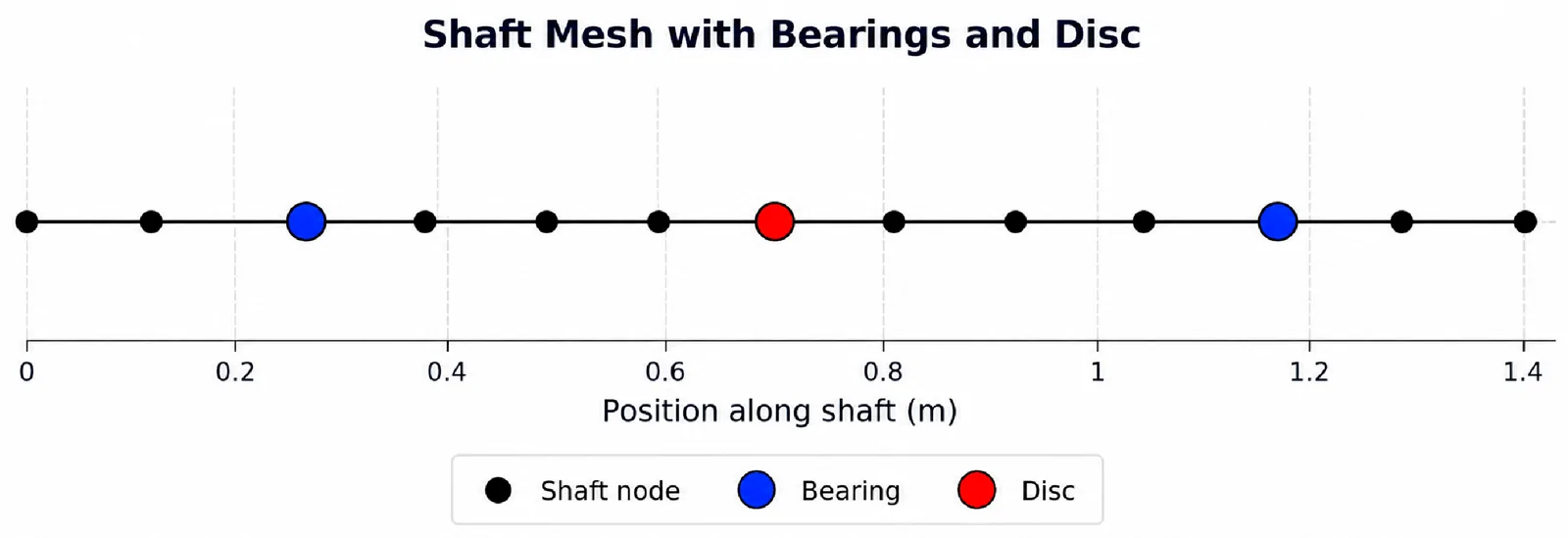
Nodes at the shaft.

**Figure 7:**
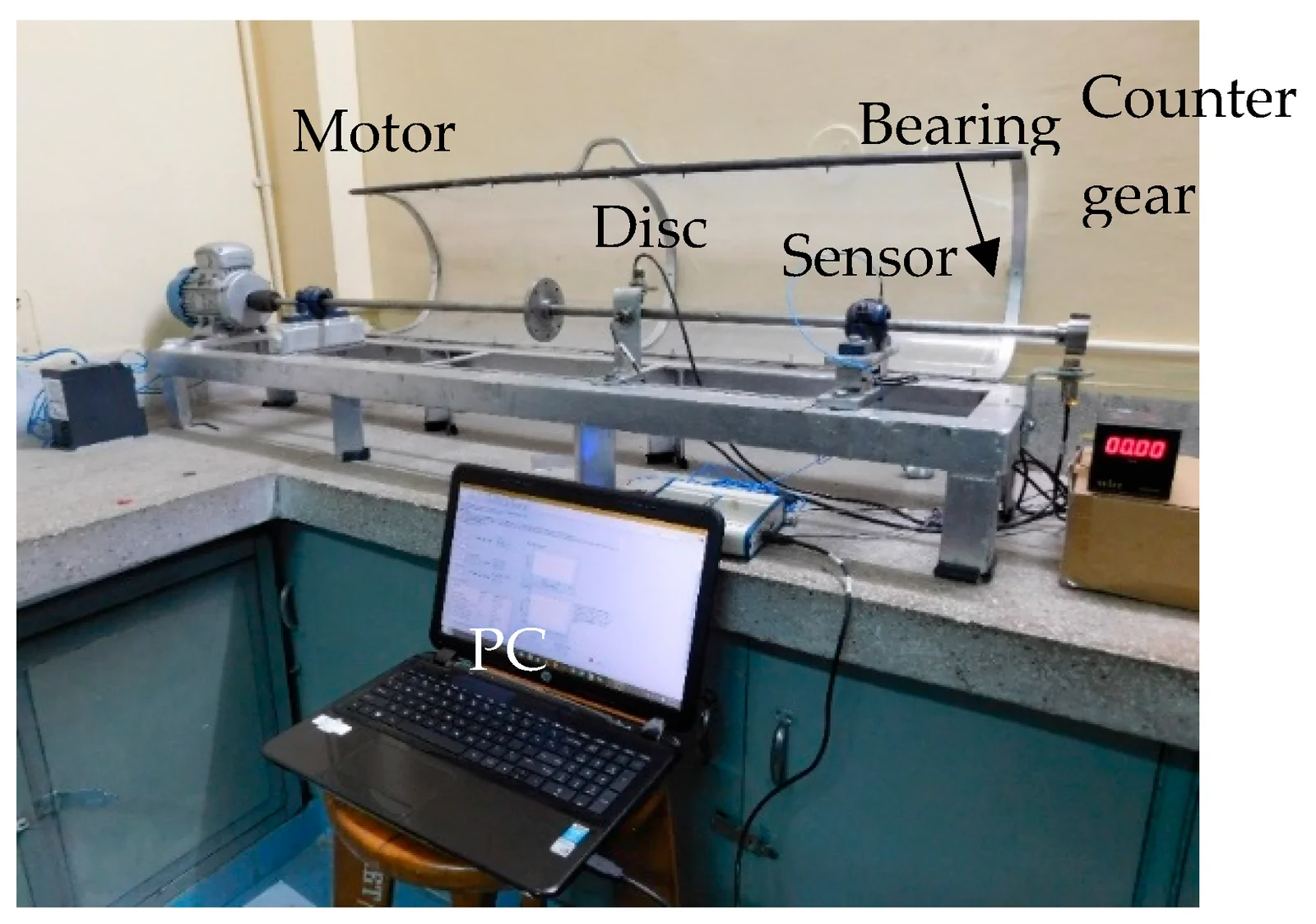
Rotor-bearing arrangement.

The resulting Figure 6 represents the arrangement of the disc and bearings. Also, Figure 7 shows the rotor bearing arrangement. The shaft covers a total length of 1.4 m and is discretised into 12 elements, resulting in 13 nodes. Further, the disc is mounted at the center of the shaft, at 0.7 m. The left bearing is positioned at node 3, and the right bearing is located at node 11.

For a particular rotor bearing system, the mass and stiffness were initially estimated using the Euler–Bernoulli beam theory. However, in actual operating conditions, the rotor often contains unknown unbalanced masses at unspecified locations. To address this discrepancy, a system identification approach was employed. This involved computing an initial guess based on theoretical estimates and refining the model using experimental results to capture the system dynamics accurately.

### 4.3. System Identification to Search Optimum Parameters for Numeric Models

**Figure 8:**
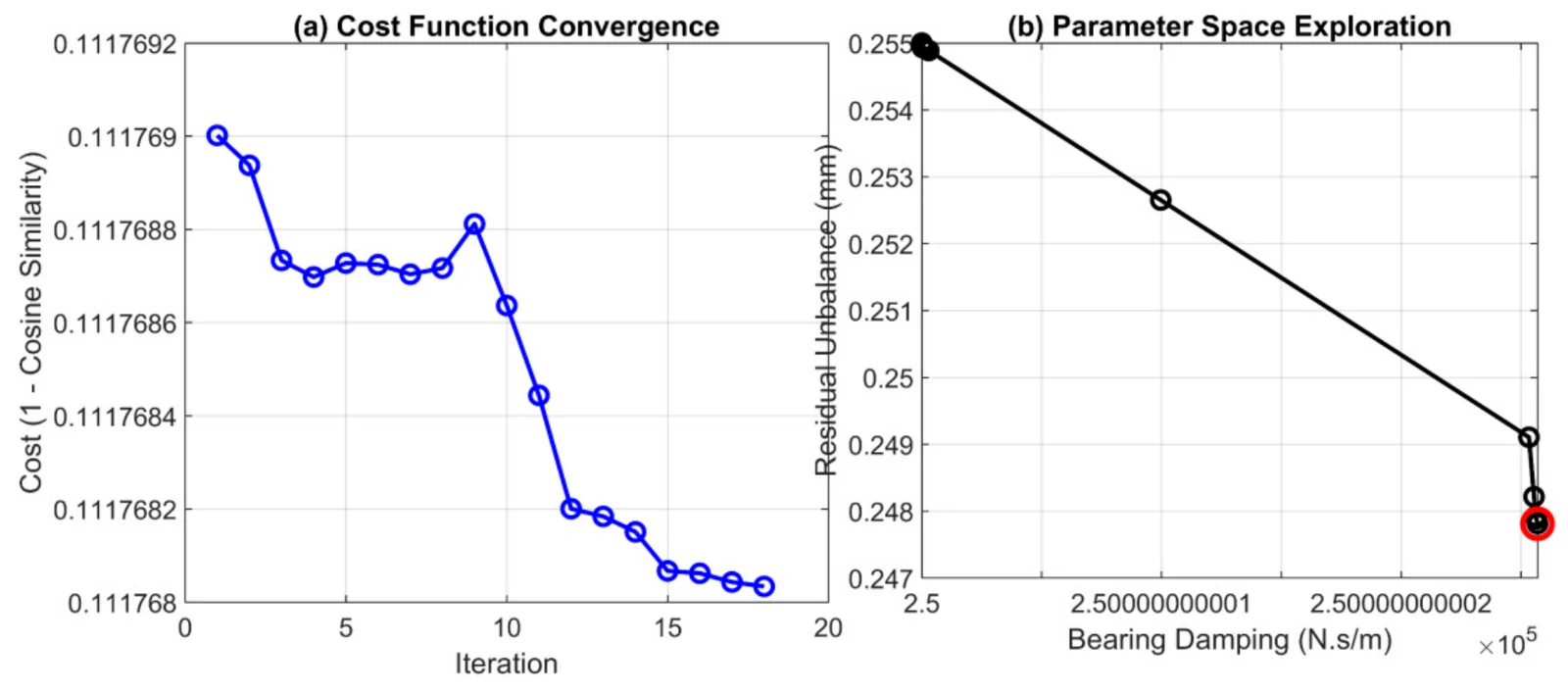
(**a**) Behavior of the cost function with respect to system identification algorithm iterations and (**b**) Parameter space exploration.

**Figure 9:**
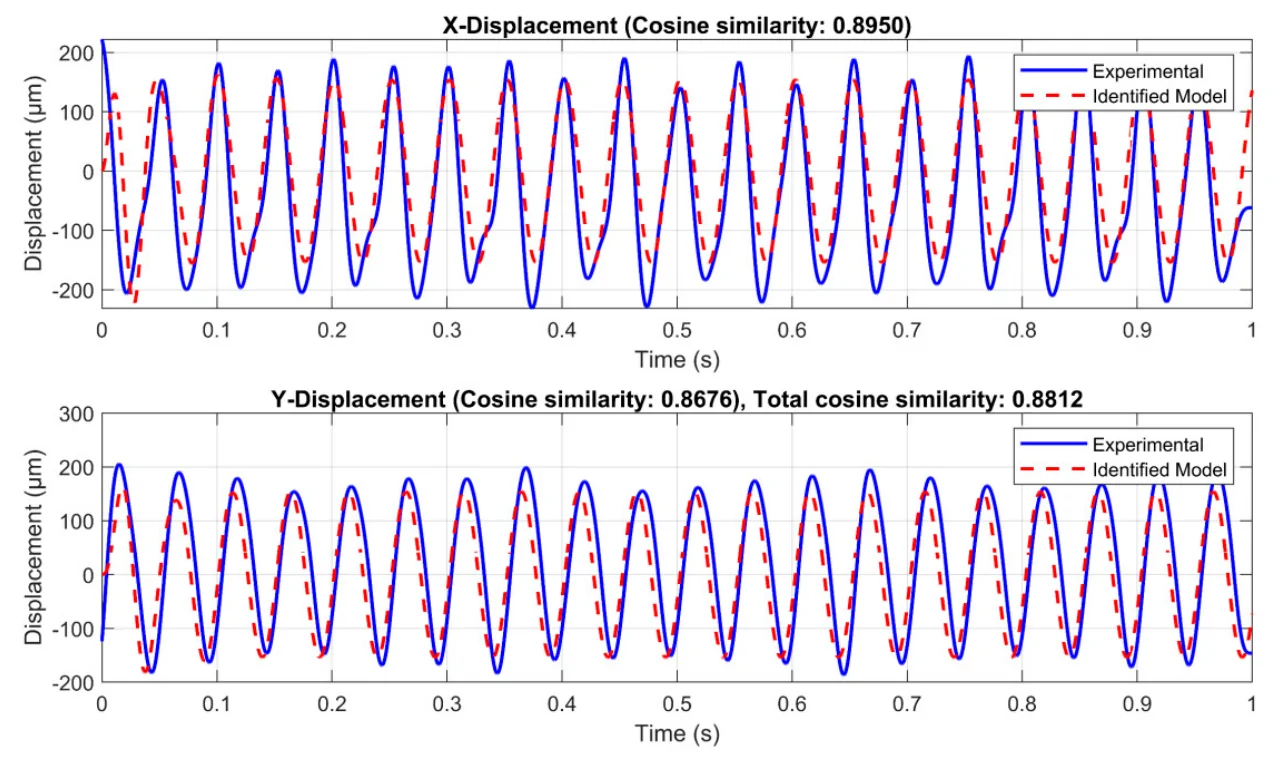
Comparison of measured and estimated responses obtained through system identification employing the Interior-Point algorithm.

The developed model simulates the rotor’s response, which is then compared with experimental measurements collected under identical conditions. The parameter estimation is performed using MATLAB’s 2020a “fmincon” command with the Interior-Point algorithm, which solves constrained non-linear problems by enforcing constraint functions and solving the Karush–Kuhn–Tucker (KKT) conditions. Optimization settings include a maximum of 500 iterations and a tolerance of 1× 10^−6^, ensuring accurate convergence. The objective, defined in Equation (13), is to minimize the discrepancy between the measured and simulated displacements by adjusting the imbalance and damping parameters. Figure 8a illustrates the behavior of the cost function with respect to this iteration. The optimization algorithm terminates after six iterations because the change in step size falls below the specified tolerance threshold. The identified bearing damping is 2.50 × 10^5^ N·s/m, and the estimated residual unbalance eccentricity is 0.0002 m, and Figure 8b shows parameter space exploration. Figure 9 compares the measured and estimated responses collected using system identification by means of the Cosine Similarity-Based Cost function (Equation (11)).

### 4.4. Defect Analysis Using Dynamic Simulation and Experimental Investigation

#### 4.4.1. Residual Unbalance Condition (Healthy Condition)

**Figure 10:**
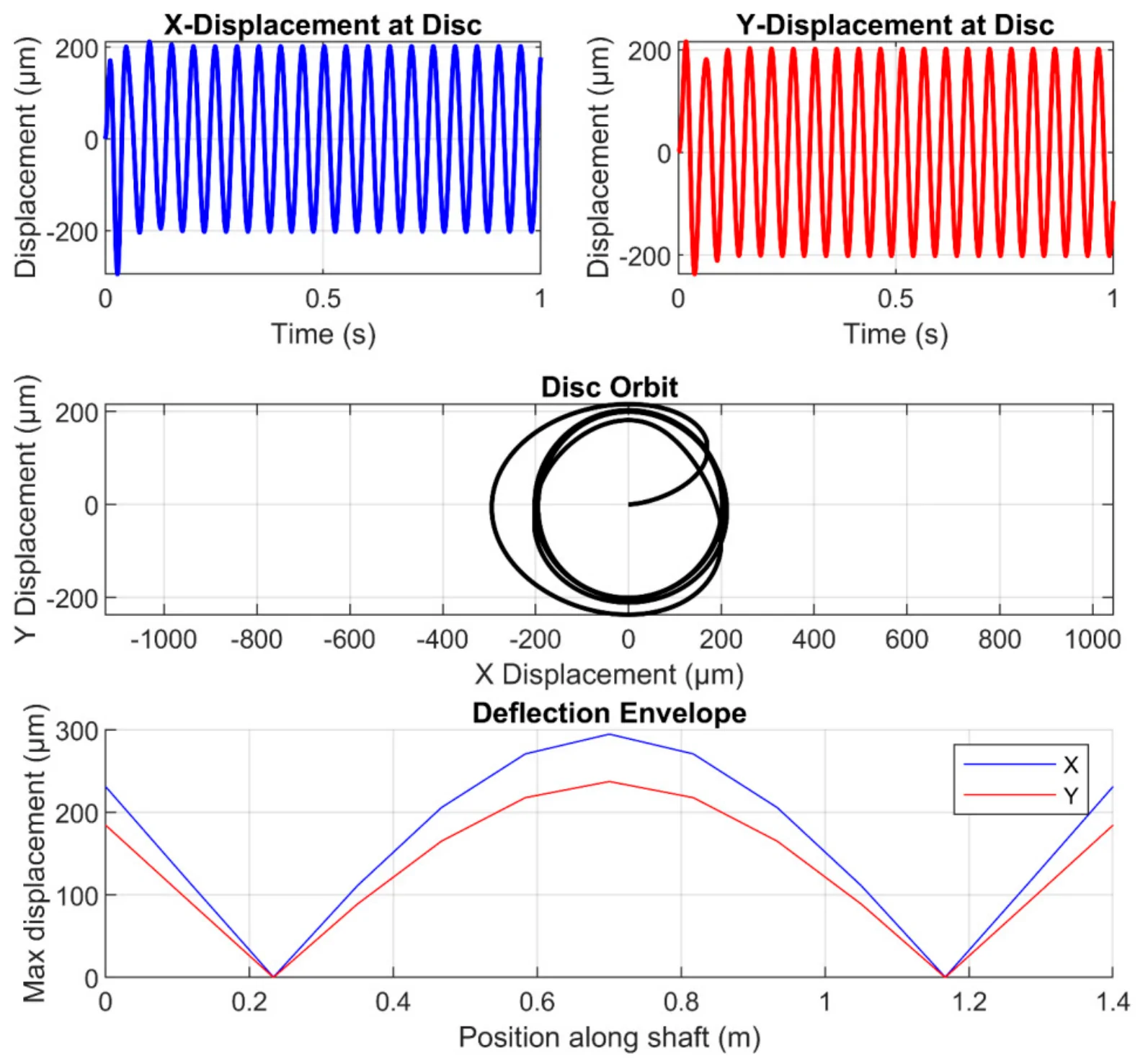
Vibration at its disc and at shaft speed of 1200 rpm (simulated response of healthy condition).

**Figure 11:**
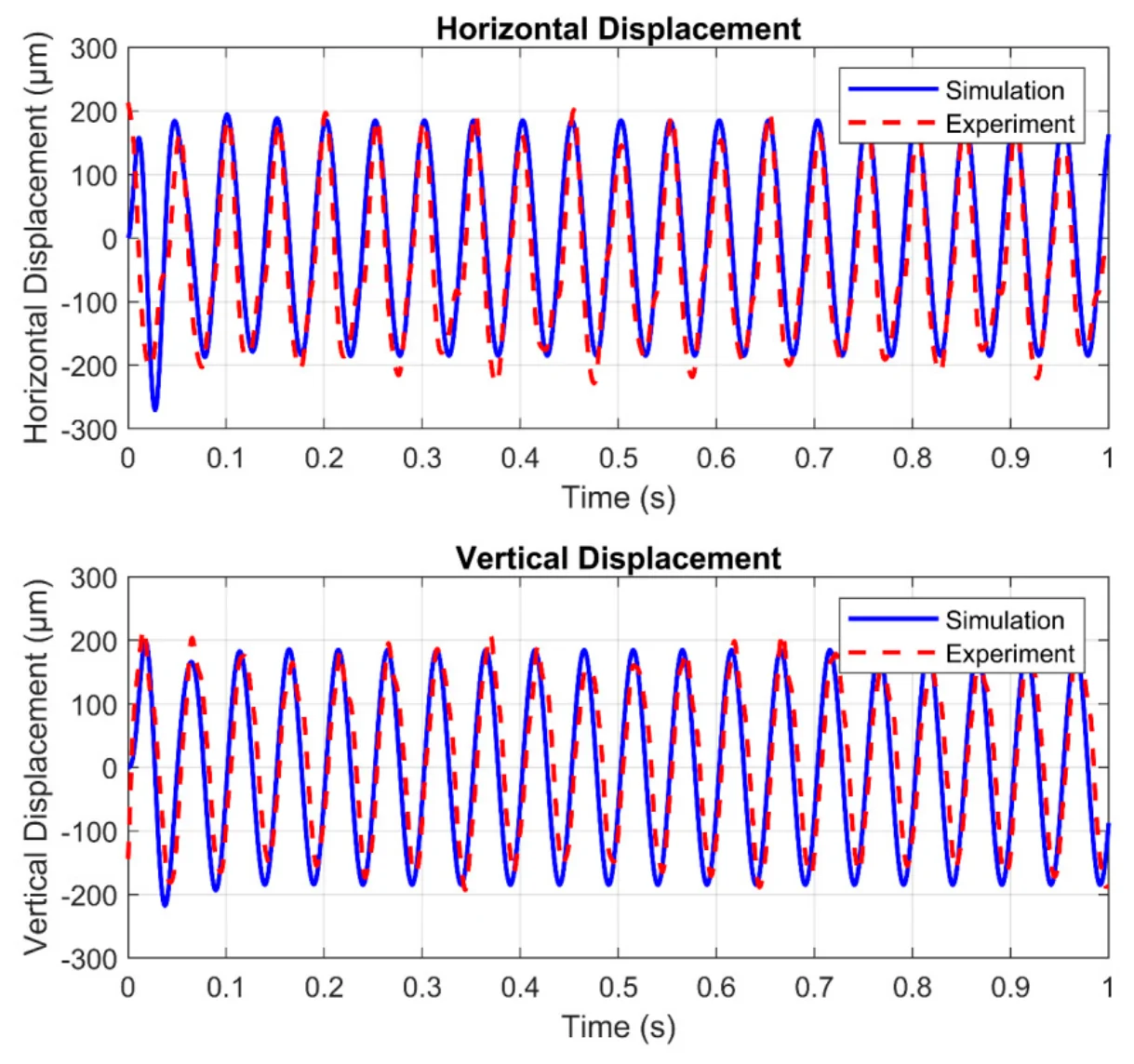
Time response of shaft displacements along the horizontal (*x*) and vertical (*y*) directions under healthy operation at 1200 rpm.

**Figure 12:**
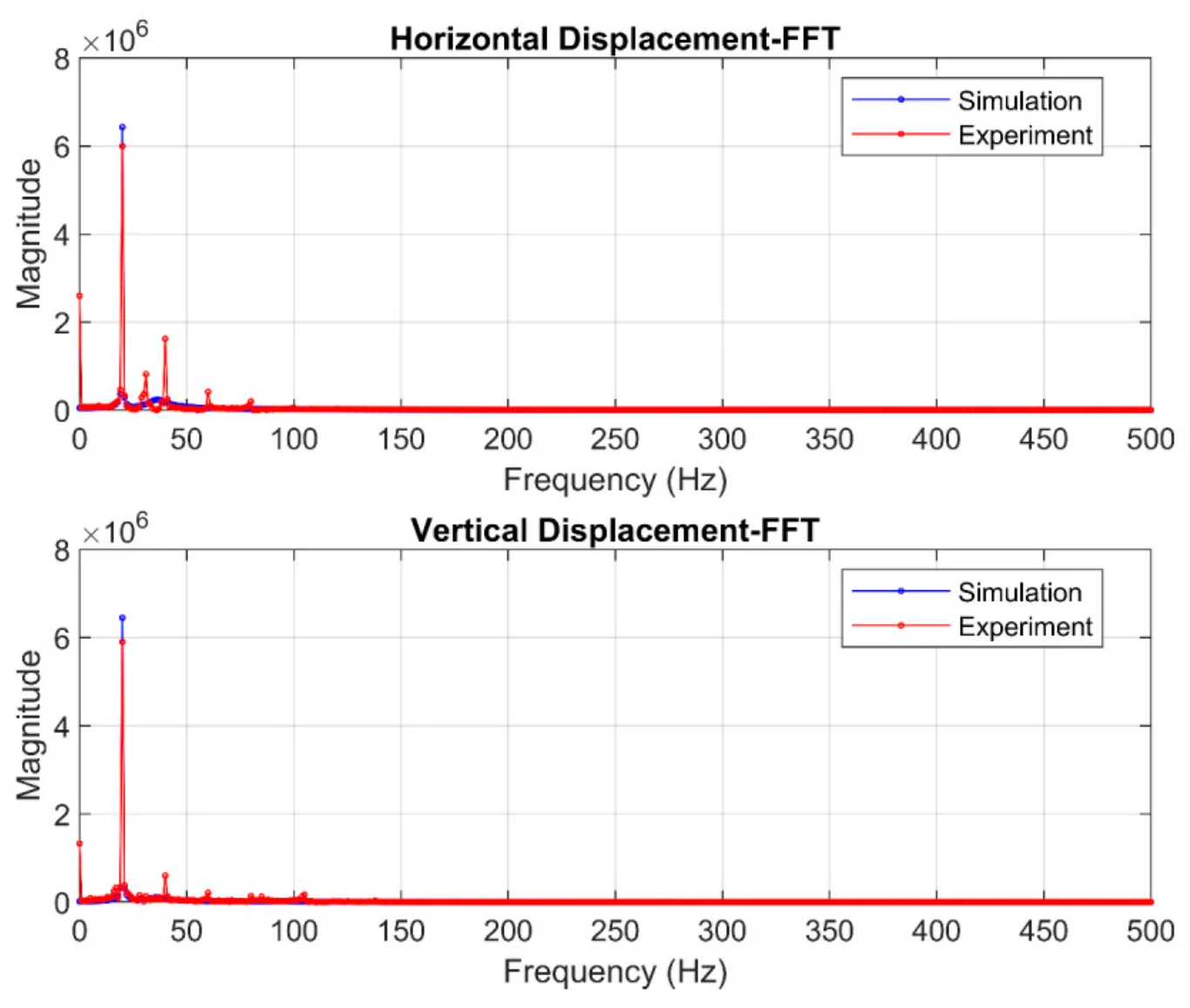
Frequency spectrum of shaft displacements in the horizontal (*x*) and vertical (*y*) directions for a healthy shaft running at 1200 rpm.

The study was conducted at a speed of 1200 rpm. Figure 10 shows the shaft vibration of the disc, its orbit, and the deflection envelope of the shaft. The excitation force due to the residual unbalance condition is shown in Equation (24). Figure 11 compares the simulated and experimental shaft displacements in the horizontal and vertical directions at 1200 rpm (20 Hz) under healthy conditions, demonstrating good agreement in vibration patterns. Figure 12 presents the corresponding FFT analysis, revealing matching dominant peaks at 20 Hz due to residual unbalance in both the simulation and experimental results, which validates the model’s accuracy for defect-free operation. The MSE (mean square error) values (Table 1) calculated between the experimental and real datasets for the horizontal and vertical datasets are 1.2× 10^−8^ and 3× 10^−9^, respectively.

(24) $$F_{ext}=\left[\begin{array}{c} m_{disc}.\omega^{2}.e.\cos\left(\omega .t\right)\\ m_{disc}.\omega^{2}.e.sin\left(\omega .t\right)-m_{disc}*\;g \end{array}\right]$$

Here, $m_{disc}$ denotes the mass of the disc located at 0.7 m, with a value of 0.5 kg; ω is the shaft’s angular velocity, equal to 125.66 rad/s; e represents the eccentricity obtained through system identification, equal to 0.0002 m; t is the time vector; and g (gravitational acceleration constant) = 9.81 m/s^2^

#### 4.4.2. Induced Unbalanced Condition

**Figure 13:**
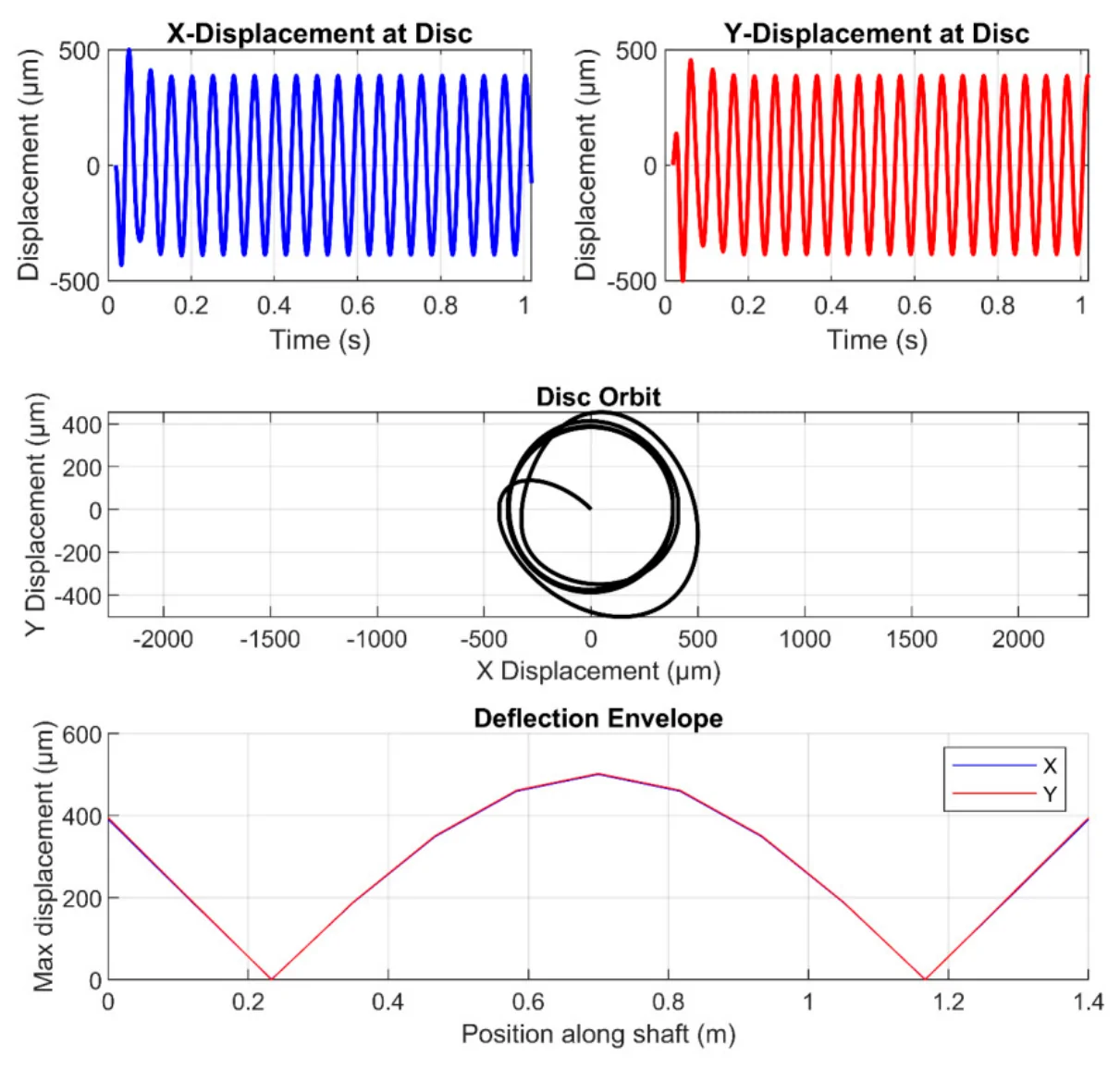
Vibration at its disc and at shaft speed of 1200 rpm (simulated response of induced unbalanced condition).

**Figure 14:**
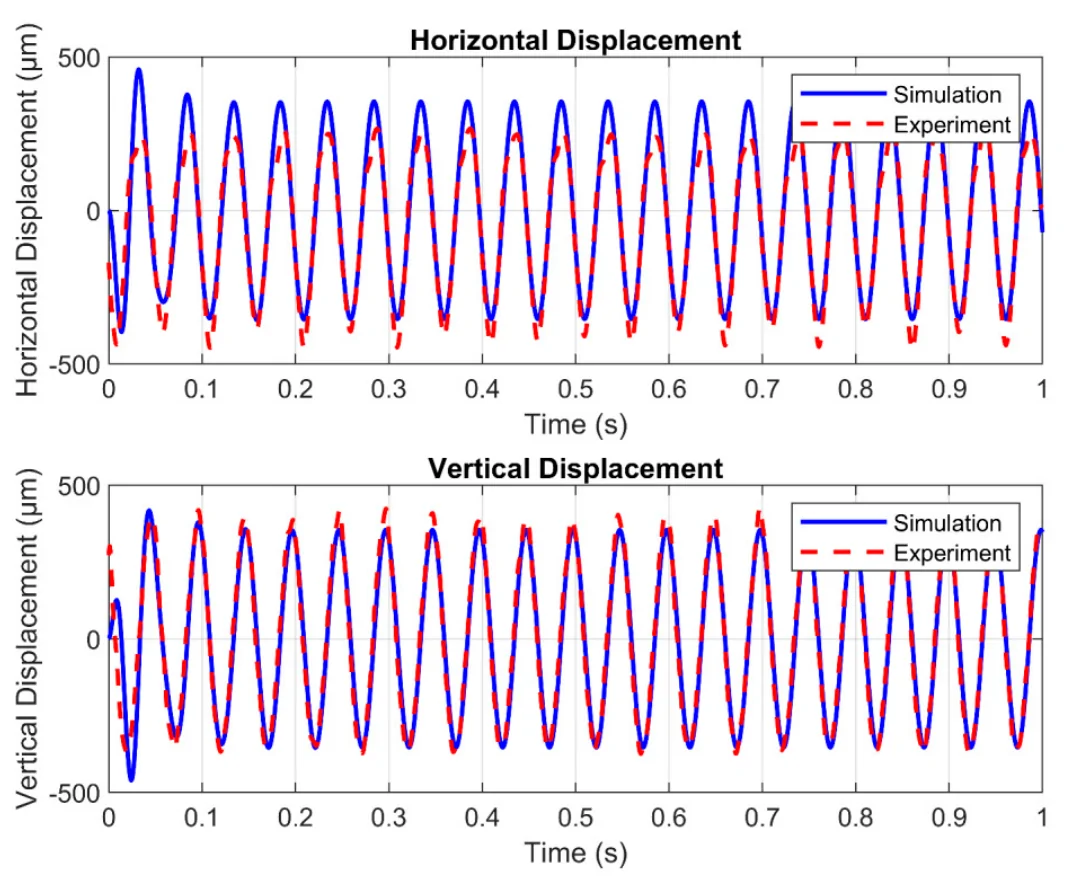
Time response of shaft displacements along the horizontal (x) and vertical (y) axes under unbalanced operation at 1200 rpm.

**Figure 15:**
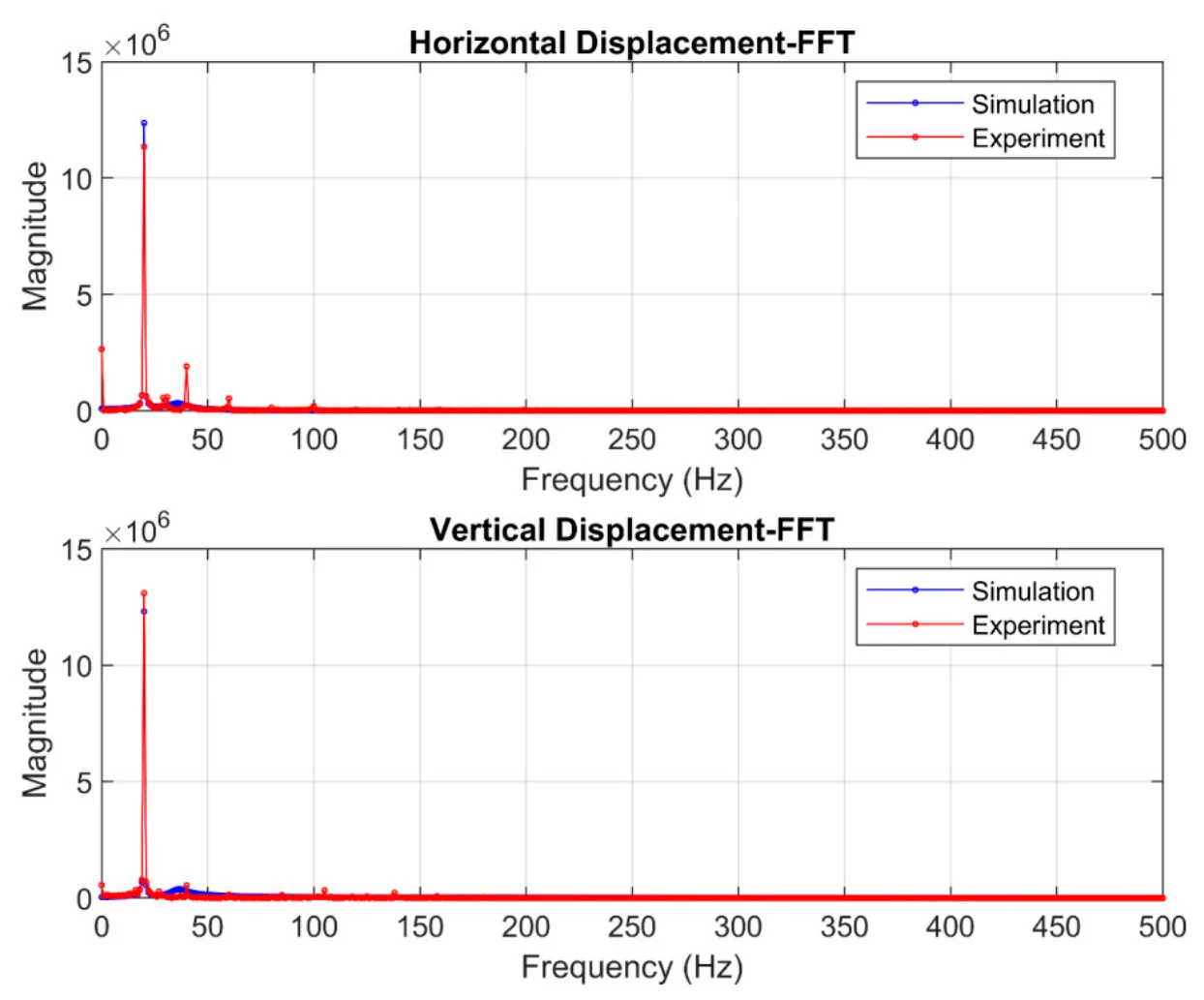
Frequency spectrum of shaft displacements in the horizontal (*x*) and vertical (*y*) directions for an unbalanced shaft running at 1200 rpm.

Figure 13 illustrates the disc’s shaft vibration, the resulting orbital trace, and the shaft’s deflection envelope. The excitation force of the induced unbalance condition is given by Equation (25). The orbit plot of the induced unbalance condition (Figure 13) is bigger compared to that of the residual unbalance condition (Figure 10). Figure 14 compares simulated and experimental shaft displacements in the horizontal and vertical directions at 1200 rpm (20 Hz) under induced conditions. The close correspondence of the two traces confirms that the model reproduces the observed vibration patterns. Figure 15 presents the FFT of these responses. Both simulation and experiment exhibit matching dominant peaks at 20 Hz, attributable to induced unbalance, which again confirms the model’s accuracy in predicting induced unbalance. The MSE between simulation and experiment is 2.6 × 10^−8^ times for the horizontal (X) data and 1.09 × 10^−7^ for vertical (Y) data.

(25) $$F_{ext}=\left[\begin{array}{c} m_{disc}.\omega^{2}.e.\cos\left(\omega .t\right)+m_{unb}\omega^{2}.{me}_{unb}.cos\left(\omega .t\right)\\ m_{disc}.\omega^{2}.e.sin\left(\omega .t\right)+m_{unb}\omega^{2}.{me}_{unb}.sin\left(\omega .t\right)-m_{disc}*g \end{array}\right]$$

$m_{unb}$ is added, unbalanced mass, equal to 9 × 10^−3^ kg, and $e_{unb}$ is the radius of unbalance equal to 0.04 m.

#### 4.4.3. Misalignment Condition

**Figure 16:**
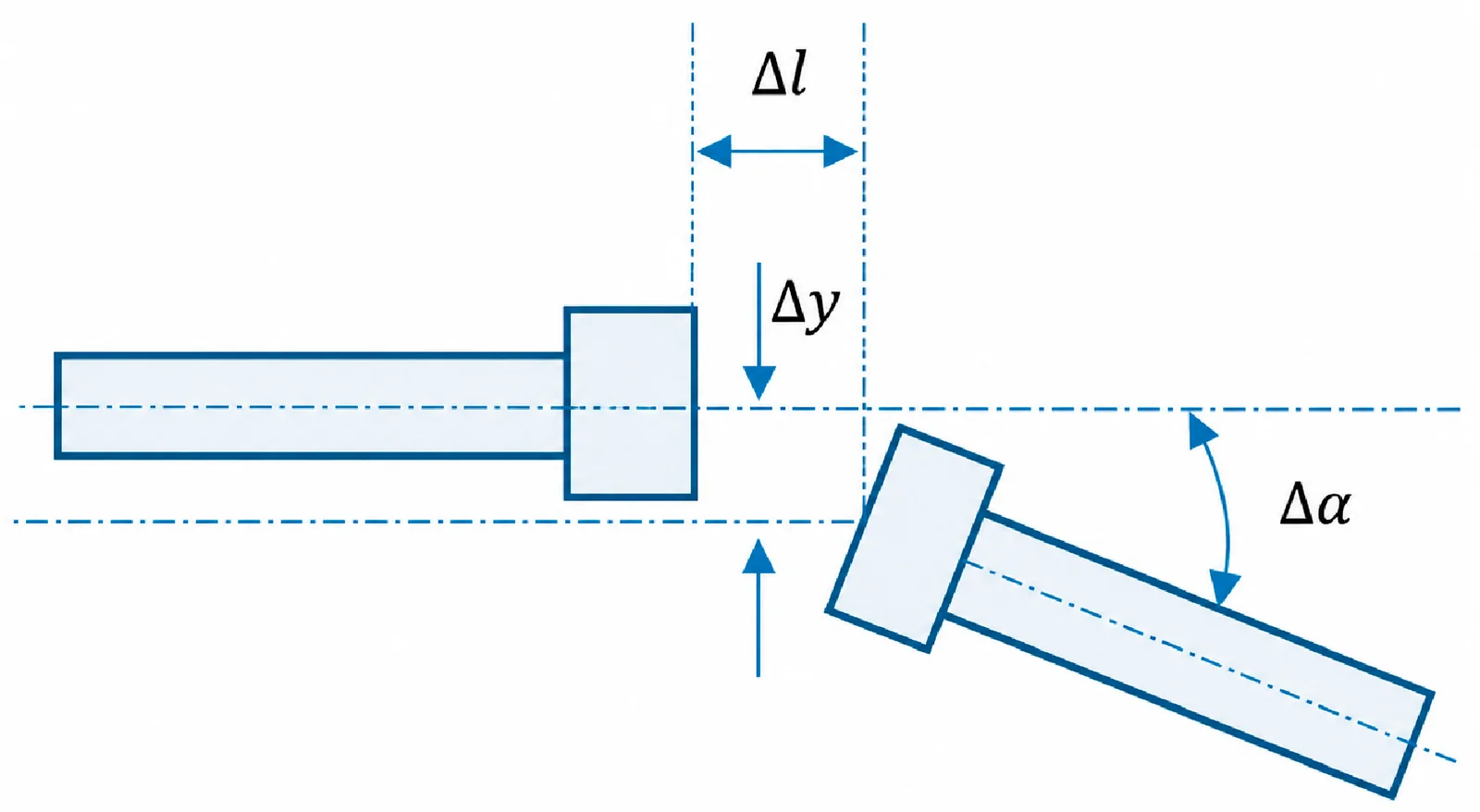
Misalignment of coupling.

**Figure 17:**
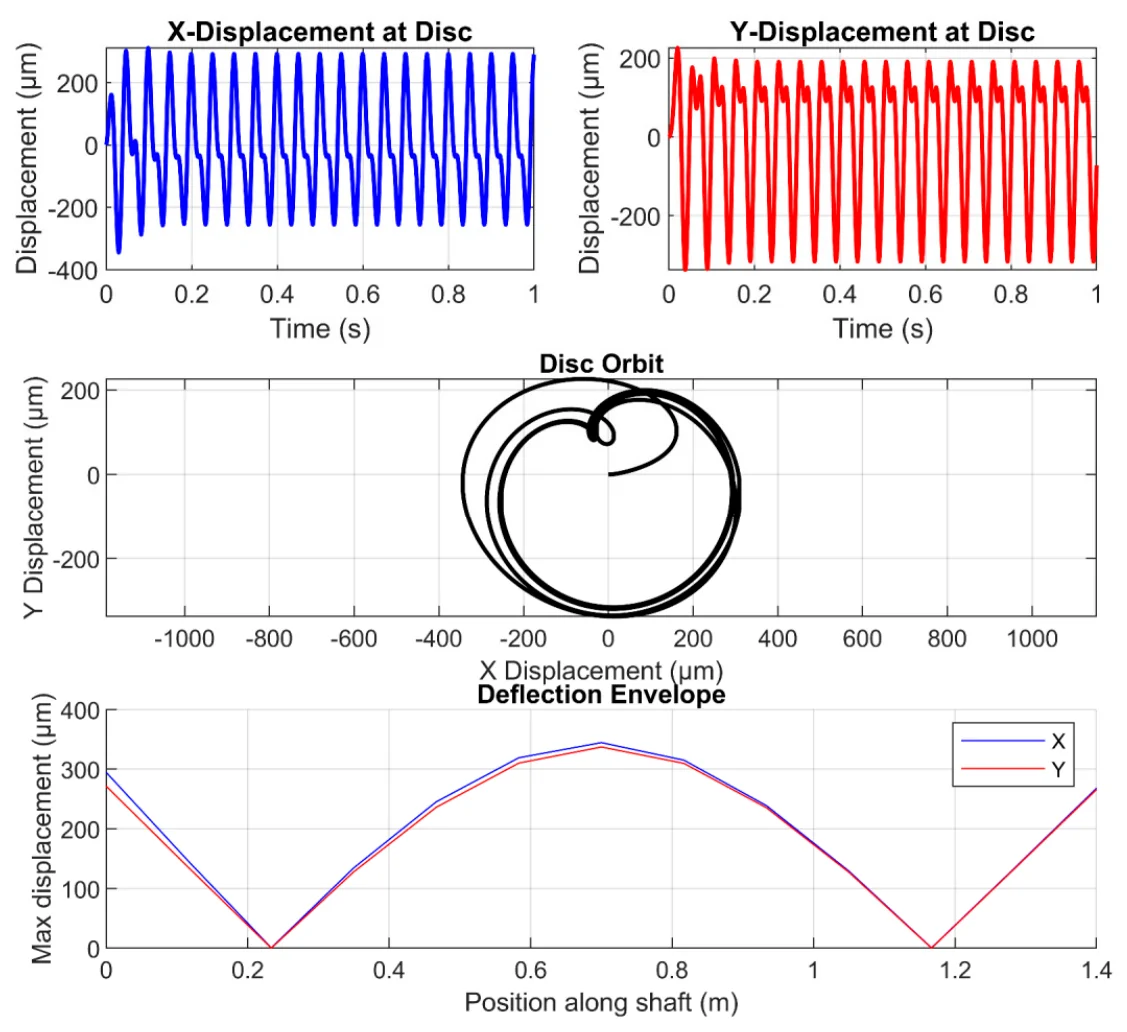
Vibration at its disc and at shaft speed of 1200 rpm (simulated response of misalignment condition).

**Figure 18:**
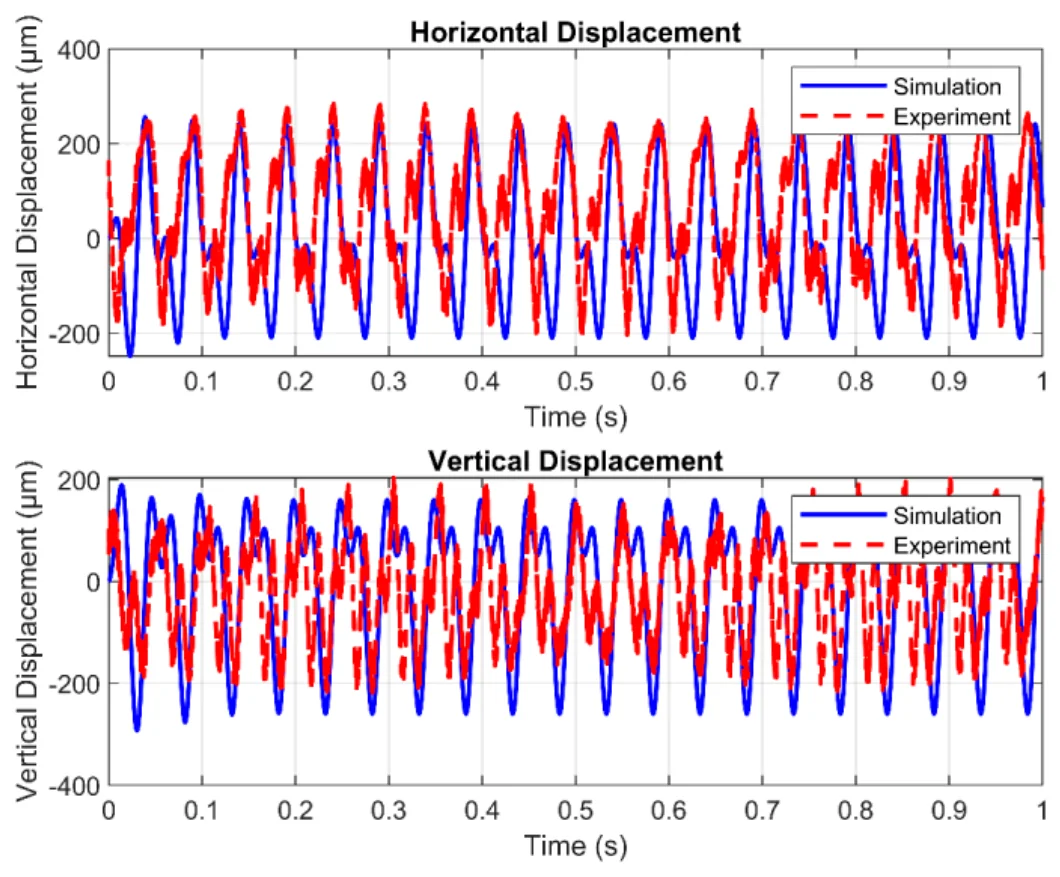
Time response of shaft displacements along the horizontal (*x*) and vertical (*y*) axes under misaligned operation at 1200 rpm.

**Figure 19:**
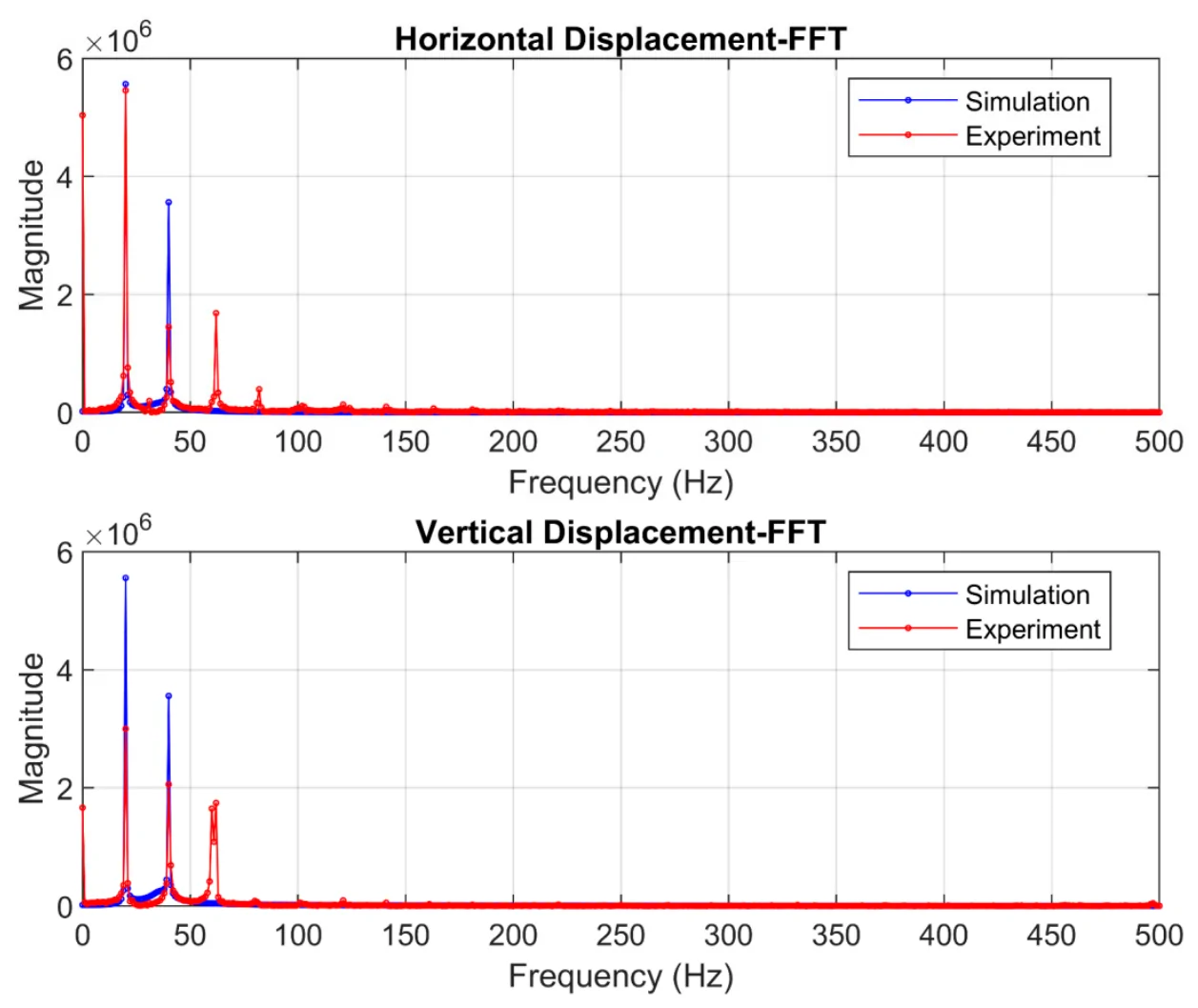
Frequency spectrum of shaft displacements in the horizontal (*x*) and vertical (*y*) directions for a misaligned shaft running at 1200 rpm.

Figure 16 illustrates the coupling misalignment, and Figure 17 illustrates the shaft vibration response of the disc, including its orbital trace and deflection envelope under induced misalignment conditions. The excitation force for the misalignment case is defined by Equation (26). The orbit plot exhibits a shape-like, mathematical character “8,” which is a typical misalignment indicator. Figure 18 compares simulated and experimental shaft displacements at 1200 rpm (20 Hz) in the horizontal and vertical directions. The close agreement between the two traces confirms that the model accurately reproduces the observed vibration behavior under misalignment conditions. Figure 19 presents the FFT analysis of these responses. The simulation and experimental results show a dominant peak at 20 Hz and a second harmonic at 40 Hz, which can be attributed to residual unbalance and misalignment. This further validates the model’s accuracy under fault conditions. The MSE between the simulation and experimental results is 1.4 × 10^−8^ in the horizontal (X) direction and 5.0 × 10^−9^ in the vertical (Y) direction.

(26) $$F_{ext}=\left[\begin{array}{c} m_{0}\left(∆y+∆l.\tan\left(\frac{∆\alpha }{2}\right)\right)\omega^{2}.cos(2\omega .t)\\ m_{0}\left(∆y+∆l.\tan\left(\frac{∆\alpha }{2}\right)\right)\omega^{2}.sin(2\omega .t)\\ \vdots \\ m_{disc}.\omega^{2}.e.cos\left(\omega .t\right)\\ m_{disc}.\omega^{2}.e.sin\left(\omega .t\right)-mdisc*g \end{array}\right]$$

where $m_{0}$ is the equivalent mass at the coupling or first node equal to 0.05 Kg.

∆y—Lateral offset due to parallel misalignment equal to 5 × 10^−3^ m.

∆l—Axial distance between misaligned shaft centres (m) equal to 0.1 m.

∆α—Angular misalignment between the shaft axes equal to 0.01 radian.

ω—Rotational speed of the shaft (rad/s).

### 4.5. Physics-Guided Modeling

The model’s detailed structure is illustrated in Figure 4. Two datasets are utilized: simulation data (referred as source domain) and experimental data (referred as target domain).

**Figure 20:**
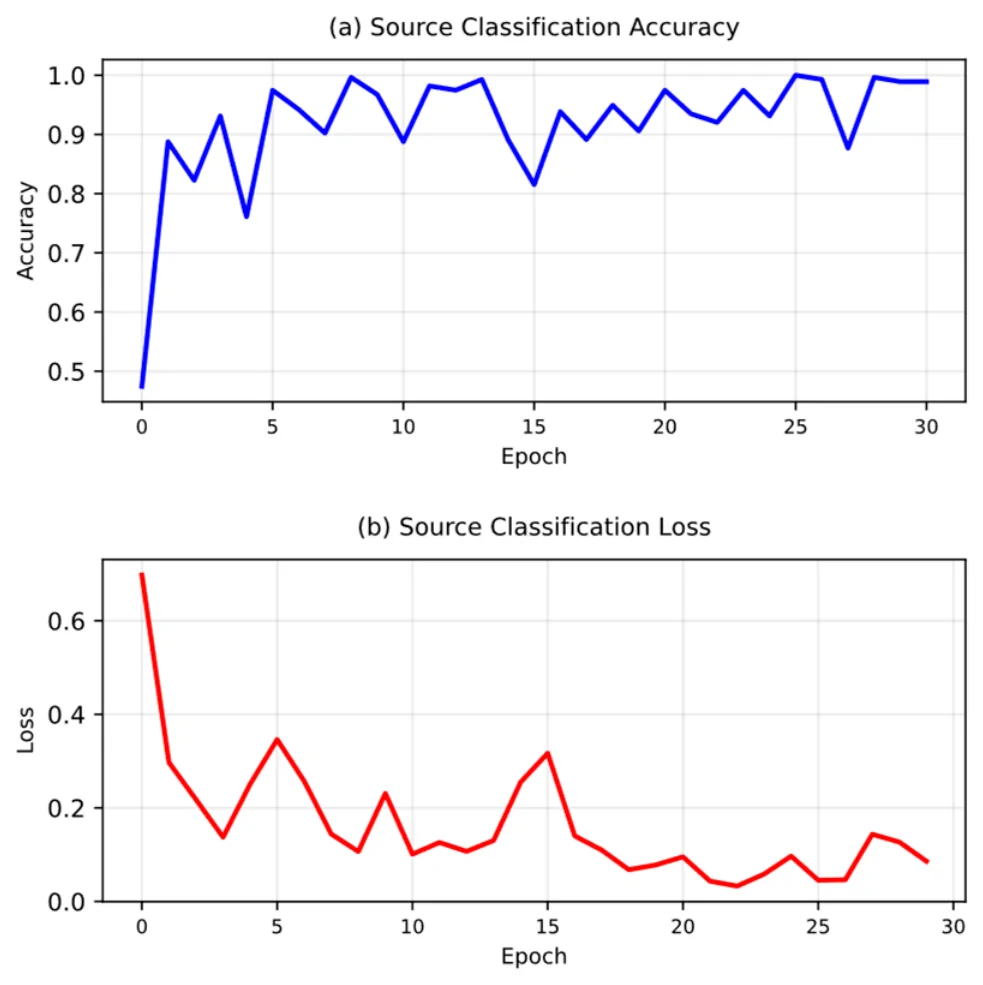
Training performance (**a**). source domain classification accuracy and (**b**) loss.

**Figure 21:**
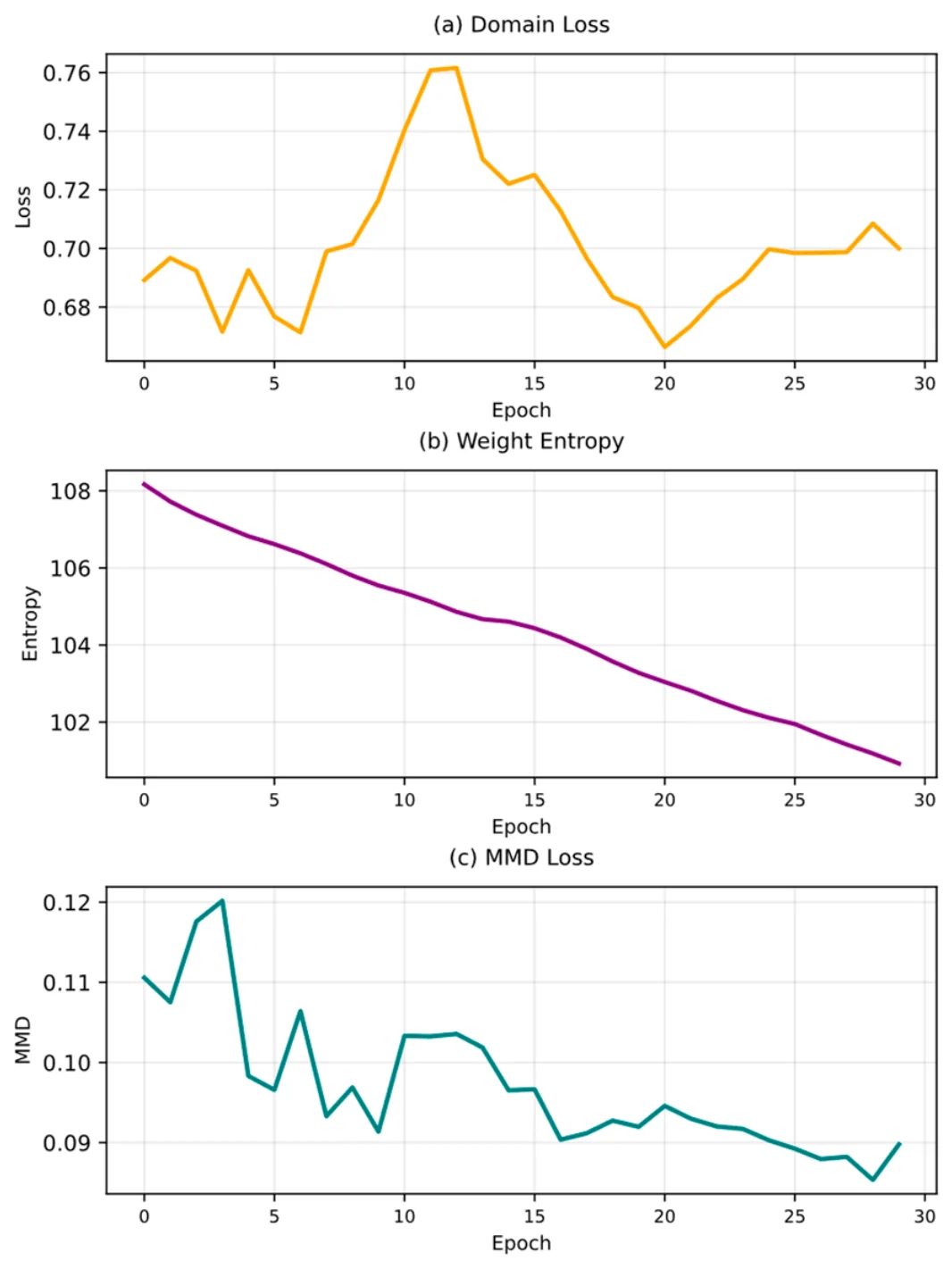
Training performance (**a**) domain loss, (**b**) entropy, and (**c**) MMD loss.

Time-series vibration signals with 1500 data points were used as input, including both horizontal (X) and vertical (Y) directions. The ResNet model extracts domain-invariant features from these signals. In order to align the source (simulated) and target (experimental) domains, adversarial training with a gradient reversal layer was applied. Figure 20a presents the classification accuracy for the source domain, while Figure 20b shows the classification loss. The total loss function includes four components: (1) classification loss, which measures prediction error (Figure 20b; (2) domain confusion loss (shown in Figure 21a; (3) weight entropy, which promotes model generalization (Figure 21b); and (4) Maximum Mean Discrepancy (MMD), which aligns feature distributions (Figure 21c).

**Figure 22:**
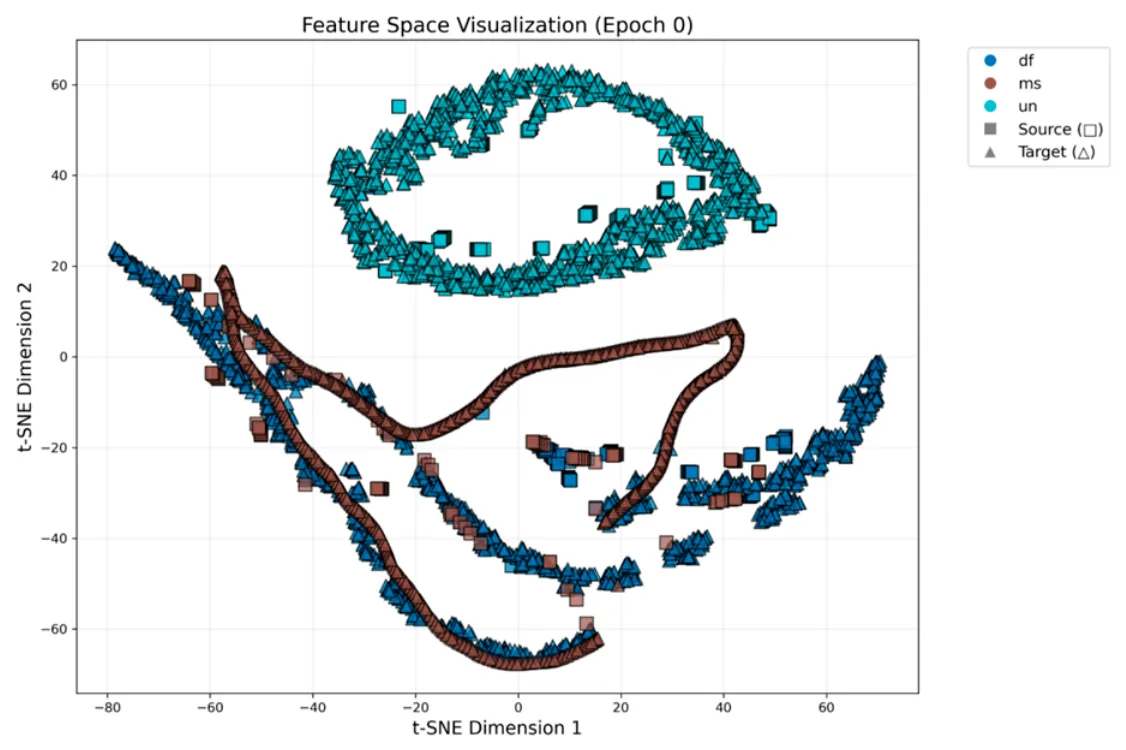
t-SNE visualization of the feature distributions at the beginning of training.

**Figure 23:**
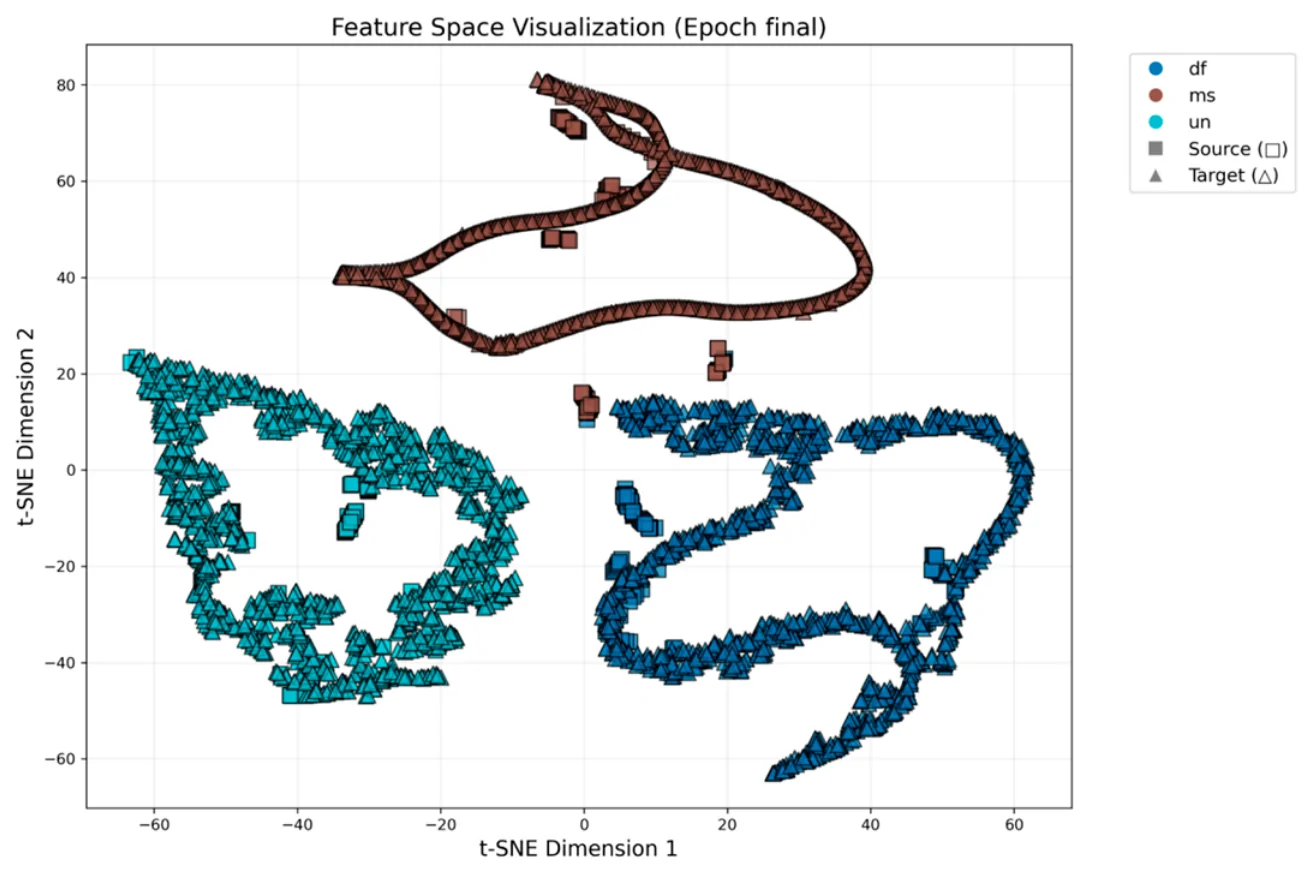
t-SNE visualization of the feature distributions at the end of training.

Training is carried out using Adam optimization (lr=0.001) with a batch size of 32. Figure 22 and Figure 23 respectively illustrate feature distributions learned by the model during training using t-distributed Stochastic Neighbor Embedding (t-SNE). In Figure 22, which represents the feature space at the beginning of training, the features from the source and target domains are separated, indicating a significant domain shift. In contrast, Figure 23 shows the feature distributions at the end of training. Here, the source and target features are more closely aligned and overlapping in the t-SNE plot. This indicates that the domain adaptation process has effectively reduced the discrepancy between the two domains.

**Ablation Study:**

Monte Carlo simulations are employed to analyze the sensitivity of hyperparameters, specifically the entropy loss weight (domain regularization ($\lambda_{1}$)) and (entropy regularization ($\lambda_{2}$)), each varied from 0 to 0.9 over 10 independent runs.

**Figure 24:**
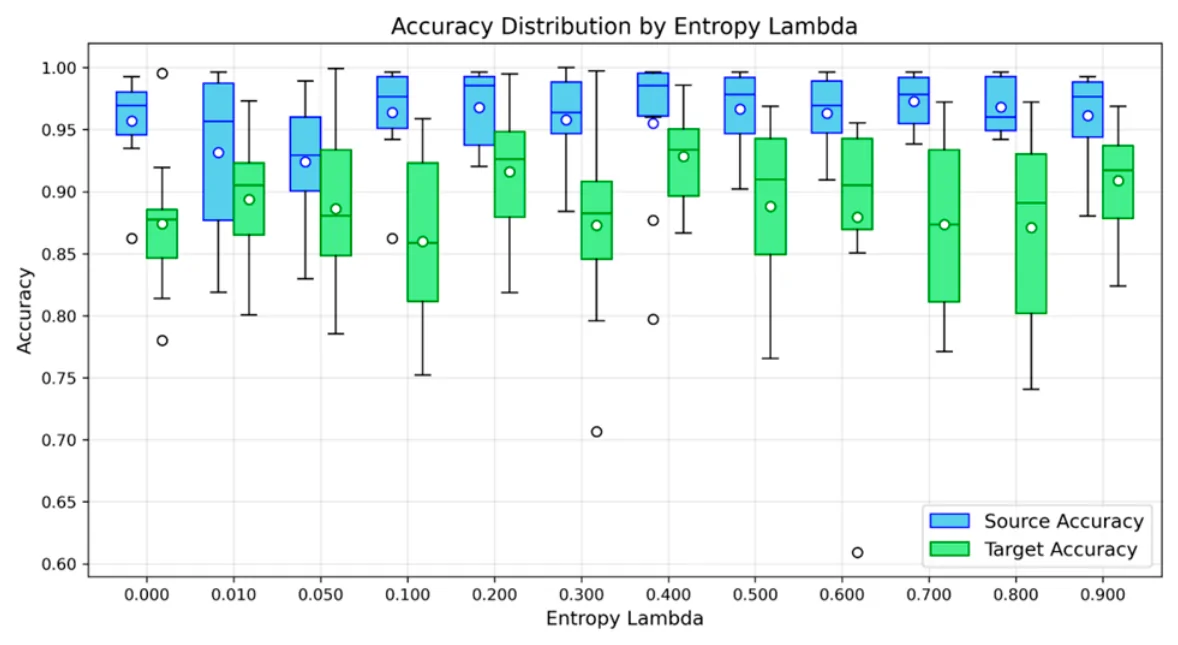
Monte Carlo simulation to see the impact of entropy regularization ($\lambda_{2}$) on the accuracy.

Figure 24 shows source and target domain accuracy as the entropy regularization ($\lambda_{2}$) is varied from 0 to 0.9, with the domain regularization ($\lambda_{1}$) fixed at 0.1. The best target domain accuracy of 93.5% is observed when entropy regularization ($\lambda_{2}$) is set to 0.4.

**Figure 25:**
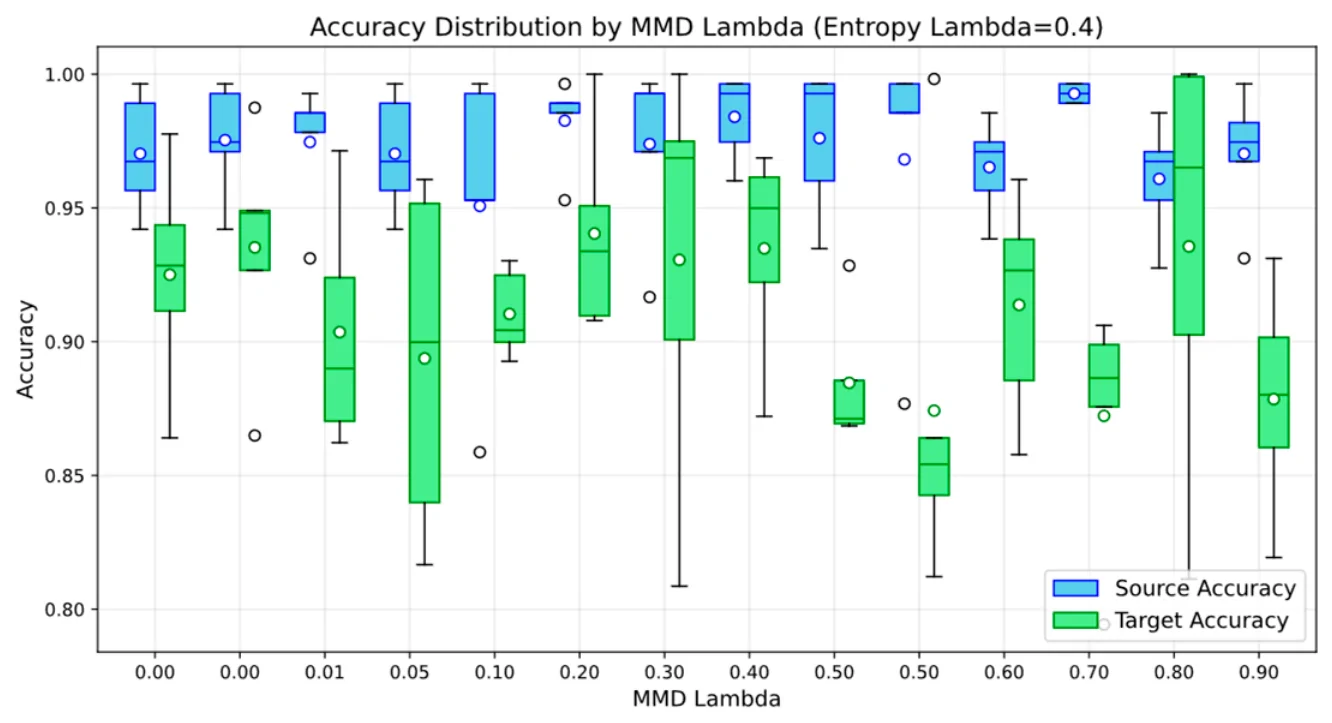
Monte Carlo simulation for observing the impact of domain regularization ($\lambda_{1}$) on the accuracy.

Figure 25 shows source and target domain accuracy as the domain regularization term ($\lambda_{1}$) is varied from 0 to 0.9, with the entropy regularization ($\lambda_{2}$) fixed at 0.4. The best target domain accuracy of 94.05% is observed when entropy regularization ($\lambda_{2}$) is set to 0.2. Table 2 presents the final accuracy for different values of the MMD regularization and fixed value of entropy regularization ($\lambda_{2}$) to 0.4.

An ablation study has been carried out (Table 3) to evaluate the contribution of each loss component in the proposed framework. The baseline ($L_{class}$) achieves a target accuracy of 76.1%, while incorporating ($L_{domain}$), $(L_{MMD}$), and ($L_{entropy}$) progressively improves the performance. The combination of ($L_{domain}$) and ($L_{MMD}$) further increases the target accuracy to 91.4%. The full model incorporating all four loss components achieves the highest target accuracy of (94.1%).

**Figure 26:**
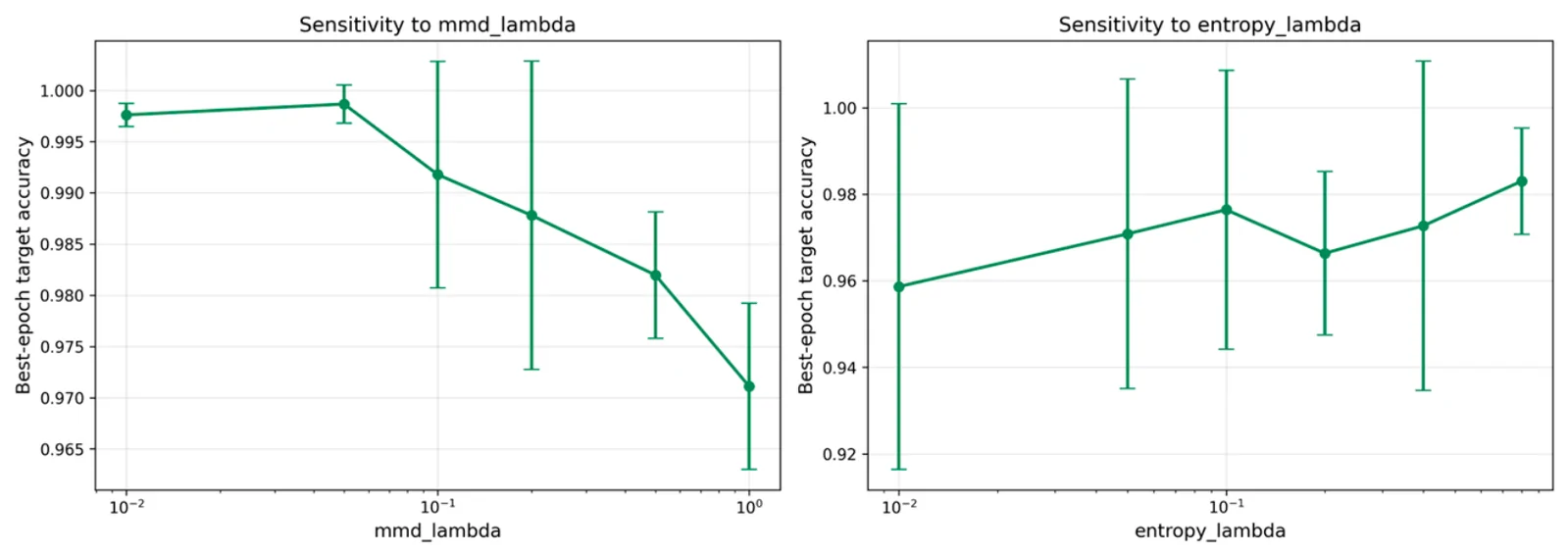
Monte Carlo simulation for observing the impact of domain regularization ($\lambda_{1}$) on the accuracy.

Figure 26 presents the sensitivity analysis of the two key hyperparameters, $\lambda_{MMD}$ and $\lambda_{entropy},\;$on target-domain accuracy. The results show that the model remains relatively stable across the tested parameter ranges, although performance varies moderately. The highest accuracy is obtained around $\lambda_{MMD}\;$= 0.03 and $\lambda_{entropy}\;$= 0.08. For larger $\lambda_{MMD}$, the target accuracy gradually decreases, indicating that excessive MMD regularization may negatively affect feature discrimination. The results demonstrate that the proposed framework is reasonably robust to variations in both hyperparameters.

**Figure 27:**
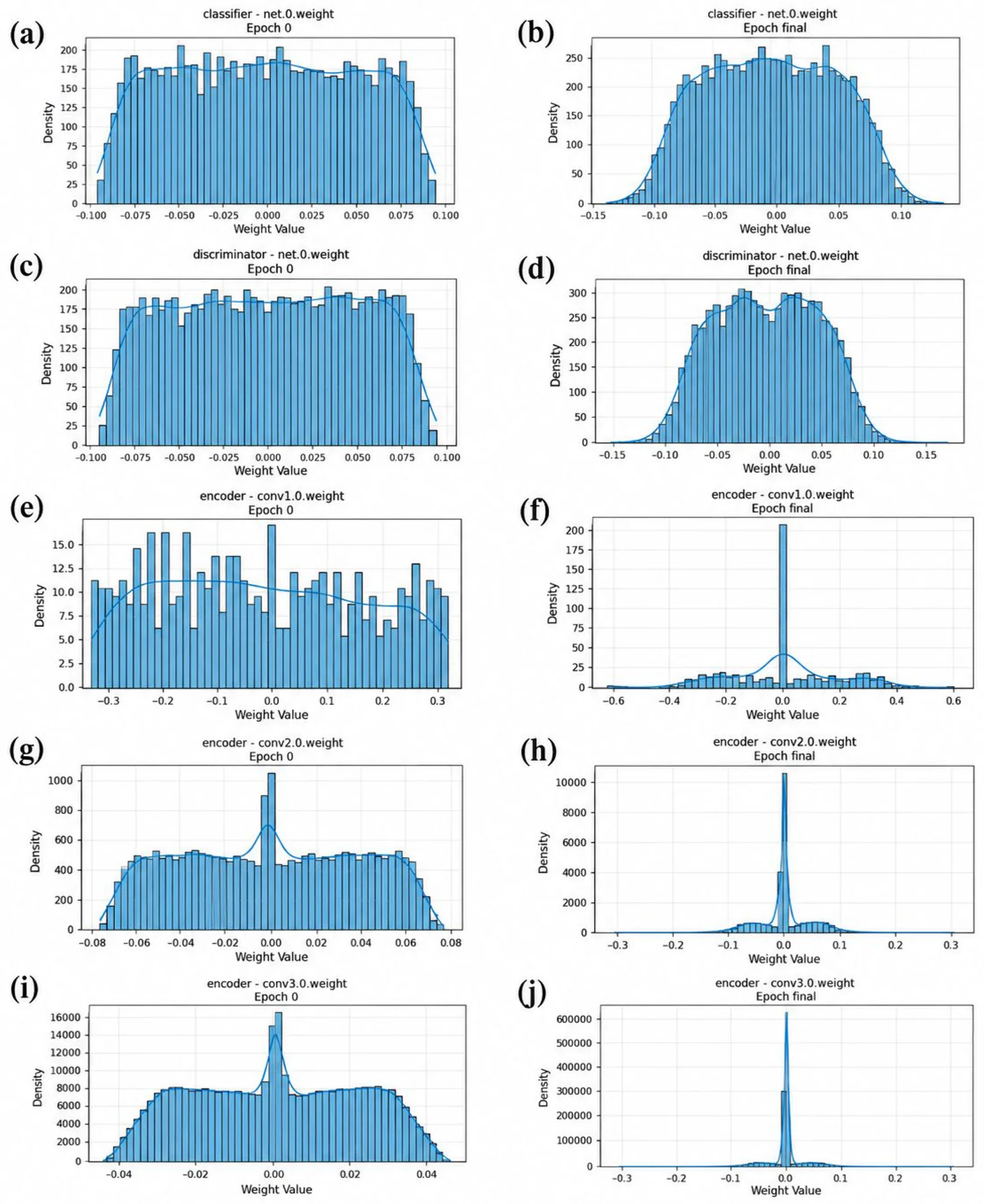
Effect of sparsity across different layers at the start of training and after training (**a**,**d**,**b**) classifier, (**c**,**d**) domain discriminator, and (**e**,**a**,**f**) convolutional layers 1 of the encoder, (**g**,**h**) convolutional layers 2 of the encoder, and (**i**,**j**) convolutional layers 3 of the encoder.

Figure 27 illustrates the effect of sparsity across different network layers at the start of training and after training. Specifically, subfigures (a) and (b) depict the sparsity patterns in the classifier layer, while (c) and (d) correspond to the domain discriminator. Subfigures (e) and (f) represent the first convolutional layer of the encoder, (g) and (h) the second convolutional layer, and (i) and (j) the third convolutional layer. These visualizations highlight how sparsity evolves during learning, providing insights into how information is selectively retained or pruned. Notably, the encoder layers exhibit more pronounced sparsity, indicating effective feature selection in the early stages of representation learning. In contrast, the classifier and domain discriminator layers remain comparatively dense, reflecting the need for richer representations at the decision-making stage.

**Figure 28:**
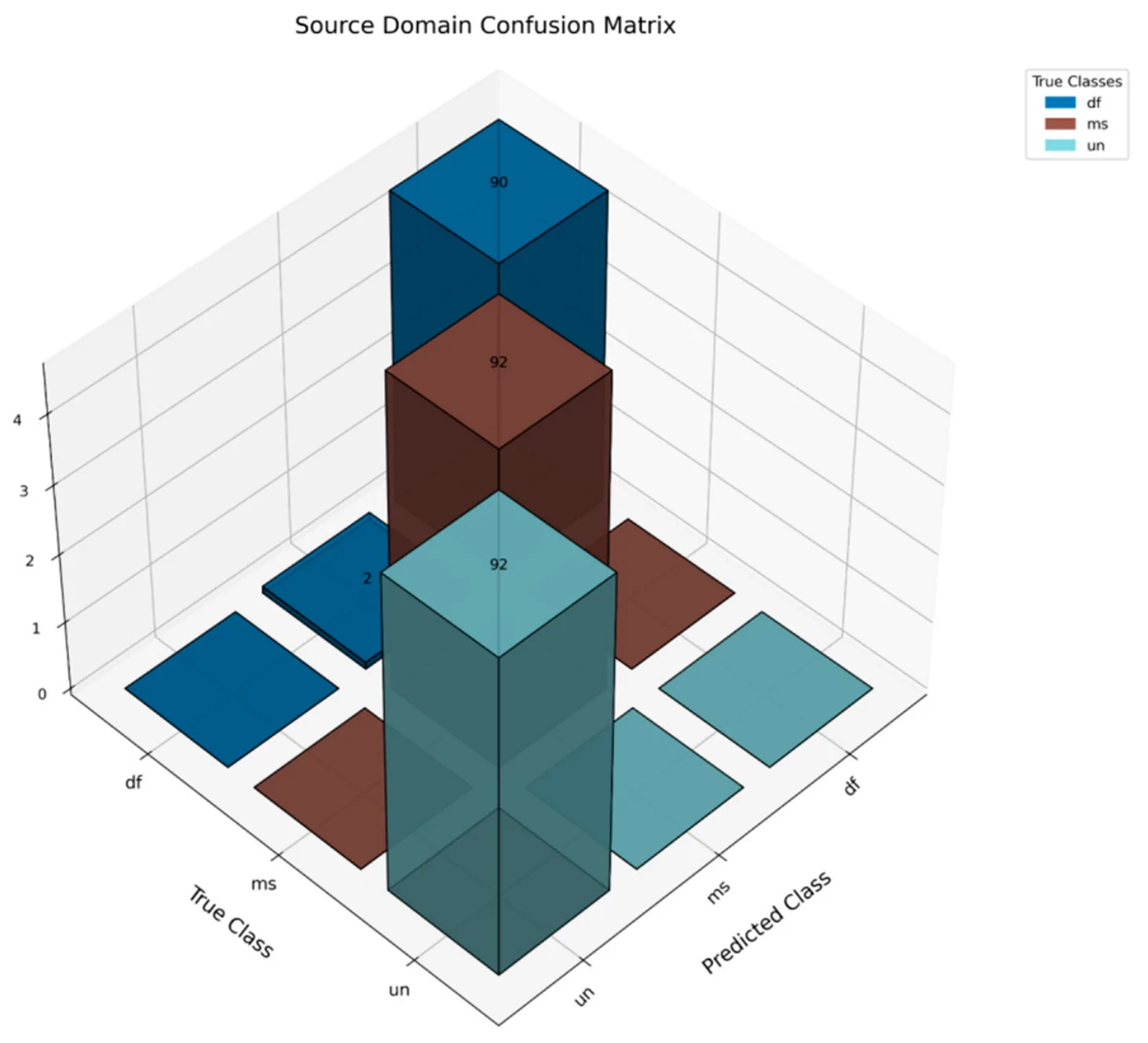
Confusion matrix (source domain).

**Figure 29:**
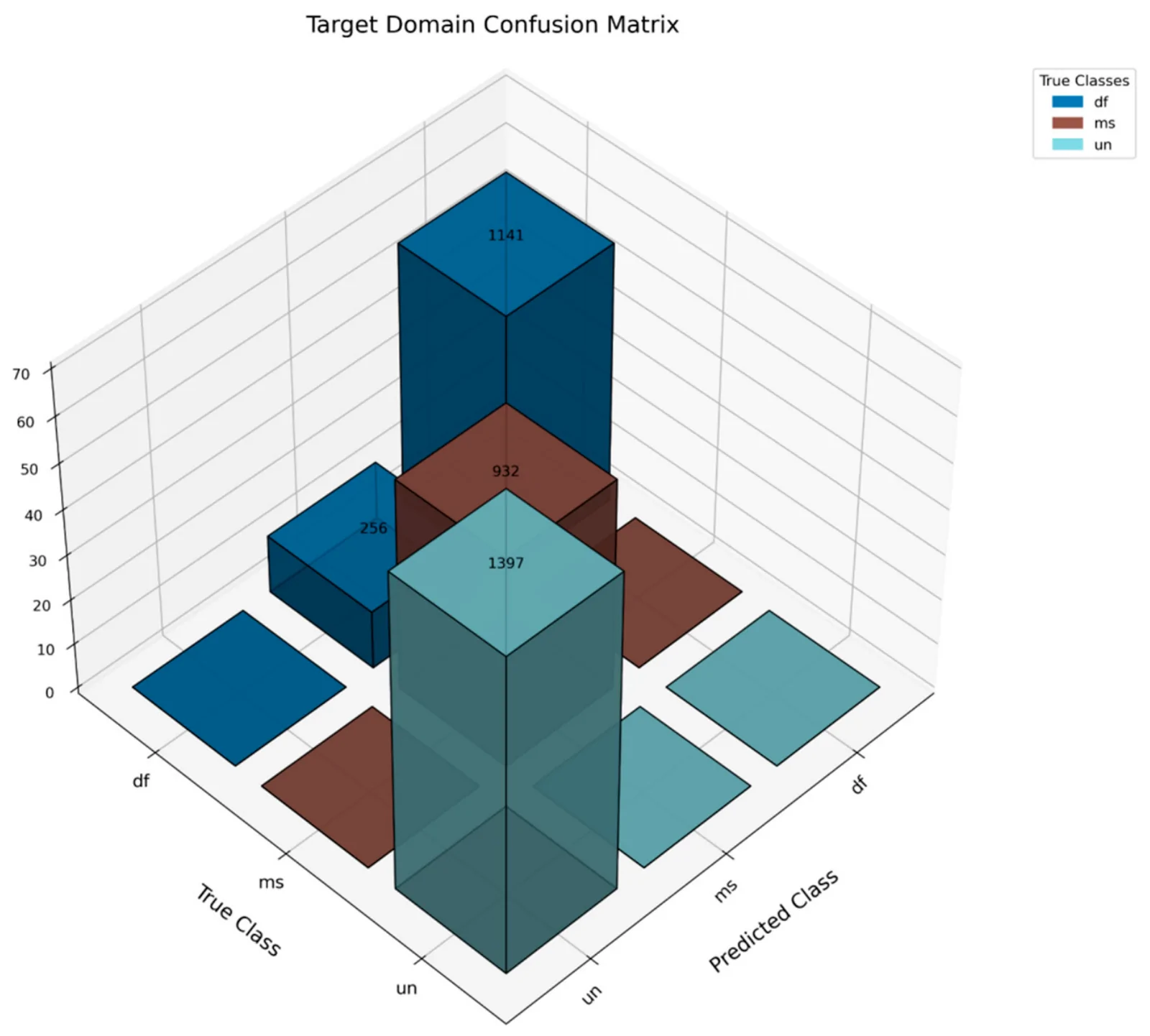
Confusion matrix (target domain).

Figure 28 presents the confusion matrix for the source domain data. Most fault types, such as unbalance and misalignment, are classified with 100% accuracy. However, the defect-free class shows a slightly lower accuracy of 97.8%. Figure 29 displays the confusion matrix for the target domain data. While unbalance and misalignment are still identified with 100% accuracy, the defect-free condition is classified with 81.67% accuracy. Notably, these results are quite promising, considering that no labeled data from the target domain was utilized during training; yet, the model demonstrates strong generalization to experimental conditions.

### 4.6. Comparison with the State-of-the-Art Method

**Figure 30:**
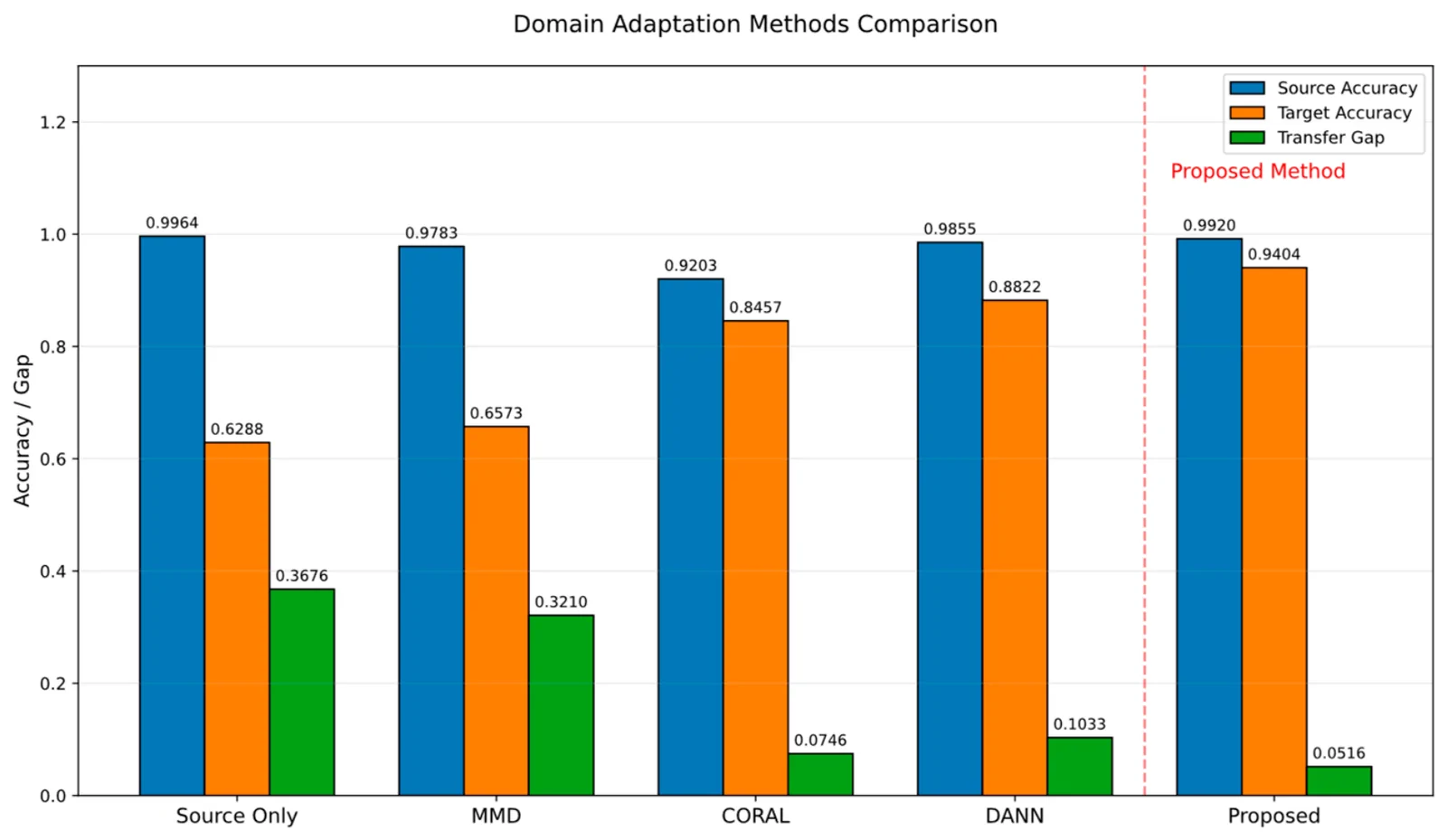
Domain adaptation performance comparison.

The resulting Figure 30 presents a comparative analysis of five domain adaptation methods: (1) Source Only, (2) MMD [44,45], (3) CORAL [46,47], (4) DANN [48], and (5) the Proposed sparsity-based domain method. These methods were chosen to represent statistical distribution matching, second-order feature alignment, and adversarial domain adaptation, respectively, providing representative benchmarks for evaluating the proposed sparsity-based domain-adaptation approach. In the source domain, accuracy indicates how effectively the model captures the training distribution, while target domain accuracy reflects its capability to generalize under distributional shift. The transfer gap, defined as the difference between these accuracies, quantifies the degradation due to domain mismatch. The proposed framework attains a target accuracy of 94.04% with a source accuracy of 99.20%, resulting in the smallest transfer gap of 5.16%, which confirms its strong domain adaptation capability. Conversely, the source only model, although achieving high source accuracy, exhibits a severe gap of 36.76%, demonstrating the adverse effects of domain shift in the absence of adaptation. Among comparative baselines, DANN provides the best performance, reaching a target accuracy of 88.22% with a 10.33% gap, while CORAL and MMD deliver partial improvements but still exhibit larger transfer gaps of 7.46% and 32.10%, respectively.

## 5. Conclusions

This study presents a physics-guided sparse deep learning model for rotor defect diagnosis, addressing the practical challenges posed by the limited availability of labeled experimental data. A physics-guided numerical model is constructed using system identification techniques and utilized to generate synthetic fault data under various rotor anomalies, including imbalance and misalignment. Based on the Euler–Bernoulli beam theory, a physics-informed numerical model is developed to simulate rotor dynamics under fault conditions. System identification techniques were employed to calibrate the model parameters (such as eccentricity and bearing damping parameter) and generate high-fidelity synthetic data for detecting rotor anomalies, including imbalance and misalignment. A hybrid training strategy was then employed by combining this labeled synthetic data with unlabeled experimental data, enabling the learning of discriminative features across domains. The proposed approach incorporates sparsity-based domain adaptation to overcome domain shift, integrating Maximum Mean Discrepancy (MMD) and entropy-based regularization. Including a sparsity constraint promotes compact and discriminative feature representations by penalizing redundancy and noise in the learned features. This introduced sparsity constraint improves the model’s ability to focus on invariant informative components across domains. The ResNet architecture is deployed for extracting the robust features from the vibration signals. At the same time, a Monte Carlo-based hyperparameter search is utilized to optimize the trade-offs between classification, MMD, and sparsity losses.

The effectiveness of the propounded sparsity-driven domain adaptation is validated through a comprehensive experimental evaluation. A comparative analysis with established domain adaptation strategies is presented to assess their performance. The propounded framework achieves the highest target accuracy of 94.04% and the lowest transfer gap of 5.16%, outperforming DANN (88.22%, gap: 10.33%), CORAL (91.73%, gap: 7.46%), and MMD (65.73%, gap: 32.10%). This substantial performance improvement highlights that enforcing sparsity in the feature space significantly enhances domain alignment and improves generalization to real-world rotor systems. The present study is limited by the evaluation at a restricted rotational speed and fault conditions. Future work will validate the proposed framework under diverse operating conditions, fault severities, and real industrial environments.

## Figures and Tables

**Table 1:** MSE between experimental and simulation data of different defect conditions.

Condition	MSE (*x*-Direction)	MSE (*y*-Direction)
Defect free	1.2× 10^−8^	3× 10^−9^
Unbalance (Mass 9 gm)	2.6× 10^−8^	1.09× 10^−7^
Misalignment	1.4× 10^−8^	5× 10^−9^

**Table 2:** Accuracy at different values of domain regularization ($\lambda_{1}$).

Domain Regularization $\left(\lambda_{1}\right)$	Source Domain Accuracy	Target Domain
0	0.9703 ± 0.0202	0.9250 ± 0.0375
0.001	0.9754 ± 0.0194	0.9352 ± 0.0403
0.01	0.9746 ± 0.0222	0.9036 ± 0.0400
0.05	0.9703 ± 0.0202	0.8937 ± 0.0578
0.1	0.9507 ± 0.0497	0.9104 ± 0.0146
0.2	0.9826 ± 0.0153	0.9404 ± 0.0338
0.3	0.9739 ± 0.0300	0.9306 ± 0.0693
0.4	0.9841 ± 0.0144	0.9349 ± 0.0352
0.5	0.9761 ± 0.0247	0.8846 ± 0.0228
0.6	0.9652 ± 0.0163	0.9138 ± 0.0371
0.7	0.9928 ± 0.0032	0.8723 ± 0.0404
0.8	0.9609 ± 0.0196	0.9356 ± 0.0716
0.9	0.9703 ± 0.0218	0.8785 ± 0.0370

**Table 3:** Accuracy under different configurations.

Configuration	Source Acc.	Target Acc.
Classification only ($L_{class}$)	0.914 ± 0.049	0.761 ± 0.132
Classification $\left(L_{class}\right)$ + Domain Loss ($L_{domain}$)	0.999 ± 0.001	0.817 ± 0.067
Classification ($L_{class}$) + MMD($L_{mmd})$	0.906 ± 0.124	0.874 ± 0.068
Classification ($L_{class}$) + Sparsity ($L_{entropy}$)	0.918 ± 0.087	0.860 ± 0.139
Classification ($L_{class}$) + Domain Loss ($L_{domain})$ +MMD ($L_{MMD}$)	0.972 ± 0.021	0.914 ± 0.075
Classification ($L_{class}$) + Domain Loss ($L_{domain})$ +MMD ($L_{MMD}$) + Sparsity ($L_{entropy}$)	0.990 ± 0.010	0.9410 ± 0.020

## Data Availability

Data can be obtained by requesting authors by email.
